# The utility of the surprise question: A useful tool for identifying patients nearing the last phase of life? A systematic review and meta-analysis

**DOI:** 10.1177/02692163221099116

**Published:** 2022-06-29

**Authors:** Eline VTJ van Lummel, Larissa Ietswaard, Nicolaas PA Zuithoff, Dave HT Tjan, Johannes JM van Delden

**Affiliations:** 1 Department of Intensive Care, Gelderse Vallei Hospital, Ede, The Netherlands; 2 Julius Center for Health Sciences and Primary Care, University Medical Center Utrecht, Utrecht, The Netherlands

**Keywords:** Surprise question, advance care planning, palliative care, systematic review, meta-analysis

## Abstract

**Background:**

The surprise question is widely used to identify patients nearing the last phase of life. Potential differences in accuracy between timeframe, patient subgroups and type of healthcare professionals answering the surprise question have been suggested. Recent studies might give new insights.

**Aim:**

To determine the accuracy of the surprise question in predicting death, differentiating by timeframe, patient subgroup and by type of healthcare professional.

**Design:**

Systematic review and meta-analysis.

**Data sources:**

Electronic databases PubMed, Embase, Cochrane Library, Scopus, Web of Science and CINAHL were searched from inception till 22nd January 2021. Studies were eligible if they used the surprise question prospectively and assessed mortality. Sensitivity, specificity, negative predictive value, positive predictive value and c-statistic were calculated.

**Results:**

Fifty-nine studies met the inclusion criteria, including 88.268 assessments. The meta-analysis resulted in an estimated sensitivity of 71.4% (95% CI [66.3–76.4]) and specificity of 74.0% (95% CI [69.3–78.6]). The negative predictive value varied from 98.0% (95% CI [97.7–98.3]) to 88.6% (95% CI [87.1–90.0]) with a mortality rate of 5% and 25% respectively. The positive predictive value varied from 12.6% (95% CI [11.0–14.2]) with a mortality rate of 5% to 47.8% (95% CI [44.2–51.3]) with a mortality rate of 25%. Seven studies provided detailed information on different healthcare professionals answering the surprise question.

**Conclusion:**

We found overall reasonable test characteristics for the surprise question. Additionally, this study showed notable differences in performance within patient subgroups. However, we did not find an indication of notable differences between timeframe and healthcare professionals.

**What is already known about the topic?**

- The surprise question (‘Would I be surprised if this patient were to die in the next 12 months?’) is widely used to identify patients nearing the last phase of life. Earlier meta-analyses showed a sensitivity of 67.0% and a specificity of 80.2% and a pooled accuracy of 74.8%.
- The surprise question seems to perform better in cancer patients compared to other patient subgroups.
- It is suggested that doctors appear to be more accurate than nurses in recognising people in the last year of life.

**What this paper adds?**

- This study is based on 88.268 surprise question assessments and shows that the surprise question has an estimated sensitivity of 71.4% (95% CI [66.3–76.4]) and specificity of 74.0% (95% CI [69.3–78.6]). The negative predictive value of the surprise question remains high with varying mortality rates.
- Analysis of timeframe subgroups showed similar sensitivity for 6- and 12-month timeframe: 74.5% (95% CI [67.6–81.4]) and 73.4% (95% CI [68.2–78.6]) respectively. Specificity was lower for a 6-month timeframe 64.3% (95% CI [56.8–71.8]) compared to a 12-month timeframe 72.9% (95% CI [67.6–78.1]).
- A sensitivity of 83.8% (95% CI [75.6–92.0]) was observed for patients with cancer and 82.5% (95% CI [60.1–100.0]) for patients with pulmonary disease, whereas the sensitivity for the emergency department was 49.1 (95% CI [35.7–62.5]). Specificity showed less variation with values between 67.3% (95% CI [53.2–81.3]) for cancer patients and 80.0% (95% CI [60.0–99.9]) for primary care patients.
- Seven studies provided detailed information on different healthcare professionals answering the surprise question. Based on these studies we did not find an indication of notable differences between the accuracy of healthcare professionals answering the surprise question.

**Implications for practice, theory or policy**

- The surprise question has a reasonable accuracy and is therefore an appropriate screening tool to identify patients that could benefit from advance care planning.
- The surprise question should not solely be seen as an indicator of prognostication of death but rather as an opportunity for renewed attention for quality of care and shared decision making by timely initiating advance care planning.

## Introduction

Palliative care aims to improve quality of life and end of life care of patients with life-threatening illnesses and to support their families. Improving end of life care is challenging due to the unpredictable course of chronic diseases. In order to benefit from palliative care, the definition of palliative care by the World Health Organisation emphasises timely identification of patients.^1^ The surprise question was proposed by Lynn et al.^2^ as a screening method to identify patients who might benefit from palliative care. It requires the healthcare professional to answer the question: ‘Would I be surprised if this patient were to die in the next 12 months?’^2^ (or a different timeframe other than 12 months).

Two earlier meta-analyses have been performed to study the accuracy of the surprise question.^3,4^ Results from Downar et al.^3^ showed a sensitivity of 67.0% and specificity of 80.2%. White et al.^4^ showed a pooled accuracy of 74.8%. Both meta-analyses included studies with different timeframes, patient subgroups and healthcare professionals. Downar et al. included studies with a 6, 12 and 18 months timeframe but did not differentiate between timeframes in their results. White et al. included studies with timeframes of 7 days, 30 days, 6 months, 6–12 months and 12 months and stated that an increase in timeframe did not impact the diagnostic accuracy. Both meta-analyses concluded that the surprise question performs better in cancer patients compared to other subgroups. White et al. suggested that doctors appear to be more accurate than nurses in recognising people in their last year of life.^4^ However, the accuracy of the surprise question by type of healthcare professional is based on one study and more research is needed.

Many studies on the surprise question have been published in recent years, potentially giving new insights, not only into the overall accuracy of the surprise question, but also into potential differences between timeframes, patient subgroups and healthcare professionals answering the surprise question. Therefore, the aim of this systematic review and meta-analysis is to determine the accuracy of the surprise question in predicting death, investigating potential differences by timeframe, patient subgroup and type of healthcare professional answering the surprise question by answering the following questions: 1. How accurate is the surprise question in identifying patients in the last year of life? 2. Are there differences in accuracy of the surprise question between various timeframes? 3. Are there differences between patient subgroups to identify patients in the last year of life when using the surprise question? 4. Are there differences between healthcare professionals in identifying patients in the last year of life when using the surprise question?

## Methods

### Study design

This study entails a systematic review and meta-analysis of articles studying the accuracy of the surprise question. This study followed the reporting guideline of the Preferred Reporting Items for Systematic Reviews and Meta-analyses (PRISMA).^5,6^

### Data sources and search strategy

A systematic search was performed in six databases from inception till January 22nd 2021: PubMed, Embase, Cochrane Library, Scopus, Web of Science and Cumulative Index to Nursing and Allied Health Literature (CINAHL). The search terms ‘surprise question’, ‘Gold Standards Framework’ and ‘NECPAL’ were combined using the Boolean operator OR. The latter two are more elaborate tools to predict the need for end of life care that also use the surprise question^7,8^ and were added after an initial pilot search. No filters or limits were applied in the search. Details of the search strategy can be found in Appendix 1. Cross-referencing of included studies was performed.

#### Eligibility criteria

##### Inclusion criteria

Studies were included if they met the following criteria:

1. Prospective studies of any design, including non-peer reviewed publications.
2. Using the surprise question as a prognostic indicator.
3. Death as outcome.

##### Exclusion criteria

Studies were excluded if they met the following criteria:

1. Text was not in English.
2. Design was retrospective.
3. Reversed surprise question (‘Would I be surprised if this patient were still alive in 12 months?’) was used.
4. The results were not obtainable from the text or after contact with the corresponding authors.
5. Timeframe of surprise question and follow-up did not match (e.g. a surprise question timeframe of 6 months and follow-up ‘this admission’).

### Study selection

Two reviewers (EvL and LI) independently screened all studies by title and abstract to identify potentially relevant studies. Subsequently full texts of the remaining studies were assessed by the same two reviewers. Screening of the studies was performed using Rayyan.^9^ Disagreements were resolved by discussion until consensus was reached. In case of doubt a third reviewer was consulted (JvD). In case of non-peer reviewed publications, databases were searched for full text versions and requested by contacting the corresponding author. In case of incomplete data or if interpretation of data was unclear, the corresponding author of (potentially) relevant studies was contacted to obtain additional data or information.

### Quality of studies assessment

The Quality in Prognosis Studies (QUIPS) tool^10^ was used for risk of bias assessment. Studies were considered of high quality if (1) the size of the eligible population and baseline characteristics were available (2) loss to follow-up was less than mortality rate and reasons for loss to follow-up were described (3) the setting and person asking the surprise question was described (4) outcome measurement was described and (5) if the risk of confounding was considered low. Studies were considered to have high confounding if decisions on limiting treatment, potentially leading to death, took place in the study setting (e.g. at the Intensive Care or dialysis unit) or when an intervention (consultation of palliative care team or advance care planning conversation) was planned based on surprise question outcome. Articles were critically appraised by two reviewers (EvL and LI). Disagreements were discussed until consensus was reached. Quality assessment did not affect the inclusion of studies.

### Data extraction and statistical analysis

Two reviewers (EvL and LI) independently extracted the following data: study population, type of healthcare professional answering the surprise question, study setting, total subjects, total surprise question assessments, surprise question timeframe, mean age, gender and mortality. A ‘no’ answer to the surprise question will be referred to as a positive answer to the surprise question, whereas a ‘yes’ answer will be referred to as negative answer to the surprise question. In studies where multiple healthcare professionals answered the surprise question, the study’s definition was used to determine whether the answer was positive (this could require consensus in case of a multidisciplinary team or require at least one healthcare professional answering ‘no’). If multiple healthcare professionals answered the surprise question and the study provided data separately, the physician’s response was used for the meta-analysis when possible. In studies where a third option for answering the surprise question besides ‘yes’ and ‘no’ was possible (e.g. ‘*unsure’)* data extraction was performed conform the study’s definition of a positive surprise question answer (e.g. ‘*unsure’* was regarded as ‘No, I would not be surprised’).

Studies were divided in subgroups based on timeframe and patient group (cancer, cardiac disease, emergency department, kidney disease, primary care and pulmonary disease). The patient groups consisting of too few studies for analysis were combined as various. If a study cohort could potentially be classified into two groups (e.g. cardiac and emergency department), the cohort was classified into the underlying organ specific disease (e.g. cardiac disease). A ‘6 to 12’ month timeframe was considered equivalent to a ‘12-month’ timeframe. In case a study contained a derivation and a validation cohort, these were counted as separate cohorts. When a study investigated two different timeframes of the surprise question, both timeframes were included in the analysis.

The accuracy of the surprise question was analysed by constructing 2 × 2 tables of the surprise question response and mortality for each study. A true positive was considered as ‘No, I would not be surprised’ and deceased within the predetermined timeframe and a true negative was considered as ‘Yes, I would be surprised’ and alive. Sensitivity, specificity, negative predictive value (NPV), positive predictive value (PPV) and confidence intervals (CI’s) were calculated for each study. CI’s were calculated with Wilson’s method.^11^ We considered for sensitivity a correct outcome corresponding to a positive surprise question answer (‘No, I would not be surprised’) patients that died during the specified timeframe, and for specificity a correct outcome corresponding to patients with a negative surprise question answer (‘Yes, I would be surprised’) that did not die during the specified timeframe. NPV represents the percentage of patients surviving when the healthcare professionals predicted survival and PPV represents the percentage of patients dying when healthcare professionals predicted death within the specified timeframe. A bivariate random effects logistic regression model was used to pool sensitivity and specificity.^12^ This model analyses the combination of sensitivity and specificity, estimates heterogeneity of sensitivity and specificity between studies and the correlation between these measures. Results from the analyses are presented as pooled sensitivity and specificity. PPV and NPV depend on prevalence of disease or mortality rate. Hence, pooled sensitivity and specificity were used to estimate pooled PPV and NPV with 95% CI for various mortality rates: 5%, 10% and 25%. From the results from this analysis, the summary c-statistic (area under the summary receiver operating characteristic curve) was estimated with formulas described by Walter.^13^ The corresponding standard error (SE) was estimated with the Delta method.^14^ The heterogeneity measure (τ^2^), differences between studies beyond the uncertainty captured by confidence intervals, was used to estimate the *I*^2^ statistic.^15^

In a second step, we assessed the impact of timeframe, patient group and peer reviewed versus non-peer reviewed studies by including these characteristics in the model. Reporting the results from the analysis with timeframe was limited to 6 and 12 months, as these were considered most relevant. We performed a likelihood ratio test to assess the influence of non-peer reviewed publications. For each subgroup we estimated pooled sensitivity, specificity, NPV, PPV and the c-statistic with CI’s. For the subgroups cardiac, emergency department and pulmonary disease, the analysis showed convergence difficulties, as the correlation between sensitivity and specificity over studies was estimated close to zero. For these analyses, we removed the correlation to obtain reliable results. Statistical analysis was performed with SAS version 9.4.^16^ Forest plots were made using Microsoft Excel version 2016.^17^

According to Dutch law, ethics approval was not required for this study.

## Results

### Study selection

**Figure 1.**
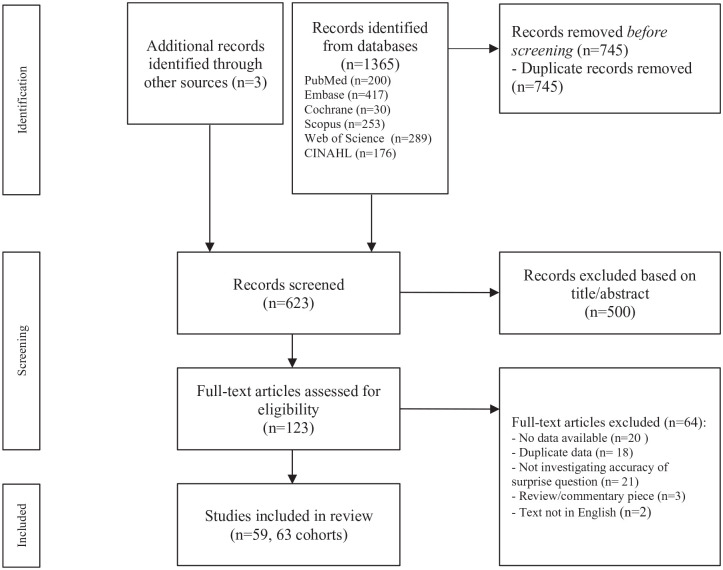
PRISMA flow diagram of screening process.^6^

The systematic search identified 1365 studies, of which 745 were duplicates. Cross-referencing resulted in the inclusion of three extra studies.^18–20^ Of the remaining 623 studies, 500 articles were excluded based on title/abstract screening. Full texts were assessed of 123 studies. Based on full text, 64 articles were excluded. In total 59 studies were included in the meta-analysis.^18,21–78^ The flowchart of the included studies can be found in Figure 1. Four studies consisted of multiple cohorts: three studies consisted of a derivation and a validation cohort^22,38,52^ and one study consisted of two different patient subgroups.^70^ In total 63 cohorts were included in our analysis. Four studies used two variants of the surprise question with varying timeframes.^31,44,64,74^

Corresponding authors of 35 potentially relevant studies were contacted in order to obtain full text or additional data in order to construct the 2 × 2 table. 18 studies were included after the authors provided additional data.^22,23,36,38,39,42,43,46,47,52,53,55,59–61,70,72,75^ Of the remaining 17 articles, two studies were excluded since they did not use the surprise question to predict death.^79,80^ Eight studies were excluded since the author was not able to not provide extra data.^81–88^ Seven studies were excluded since the corresponding author did not respond after various attempts.^89–95^ For eight other potential relevant studies (all non-peer reviewed), no contact details were available nor could these be obtained after extensive searching.^96–103^

### Study characteristics

Characteristics of included studies can be found in Appendix 2. Studies were heterogeneous in timeframe, population, setting and healthcare professional answering the surprise question (e.g. nurse v medical specialist). Most studies originated in the United States (20 studies), United Kingdom (9 studies) and The Netherlands (six studies). Forty-five studies took place in the hospital. Of these, 12 studies were performed at haemodialysis units and eight in outpatient clinics. Of the remaining 14 studies, eight took place in general practice/primary care, three in hospice care settings, one in a nursing home and one in a neurorehabilitation centre. One study took place at multiple settings (three primary care centres, one general hospital, one intermediate care centre and four nursing homes).^37^ Most studies investigated a 12-month timeframe of the surprise question (48 cohorts). Other timeframes were 3 days,^67^ 1 week,^31^ 1 month,^31,36,43,51,56,74^ 3 months,^44^ 6 months^22,38,42,46,47,53,64^ and 24 months.^57^ Four studies used two variants of the surprise question with varying timeframes.^31,44,64,74^ In general, patients included were adults (>18 years), except for one study performed in children.^44^ Eighteen studies included patients with kidney disease, 12 patients with cancer, seven with cardiac disease, seven included a diverse group of patients in general practice/primary care, six studies included patients with pulmonary disease and five studies included patients from the emergency department. In seven studies the surprise question was answered by various healthcare professionals.^26,45,50,57,60,71,75^ In two studies answering the surprise question was based on consensus of a multidisciplinary team.^30,44^ Mortality rate of all studies was on average 11.85% and varied between studies from 0.99% (primary care)^76^ to 78.78% (advanced cancer patients at the emergency department).^63^

In total five of the included studies added a third option for answering the surprise question besides ‘yes’ and ‘no’, including ‘*don’t know this patient well enough’*,^26^ ‘*don’t know’*,^66^ ‘*unsure’*,^48^ ‘*uncertain’*^49^ and ‘*defer’*.^71^ In total these answers represent 61 of 88.268 surprise question assessments, varying from 6%^48^ to 9%^71^ per study. In two studies this percentage could not be retrieved.^26,49^

### Quality assessment: Risk of bias

A detailed overview of the risk of bias assessment is presented in Appendix 3. Three studies had a high risk of bias (two non-peer reviewed), 13 studies (eight non-peer reviewed) had a moderate risk of bias and 43 studies (six non-peer reviewed) had a low risk of bias. Most methodological issues were in study population (domain 1: eight high and 30 intermediate risk of bias) and study confounding (domain 5: two high and 17 intermediate risk of bias). A risk of selection bias was in many studies caused by not specifying the eligible population. An intermediate or high-risk assessment in study confounding was in most studies due to the setting and patient population (e.g. haemodialysis patients) or caused by planning an intervention based on the outcome of the surprise question.

### Meta-analysis

In total 88.268 assessments were included from 59 different studies and 63 different cohorts. Sensitivity between individual studies varied from 12.5%^74^ to 100%,^28^ specificity varied from 26.3%^67^ to 98.6%,^76^ NPV from 35.1%^53^ to 100%^28^ and PPV from 5.4%^43,56^ to 84.7%.^63^ Individual study results and forest plots of the sensitivity and specificity can be found in Appendices 4–6. A likelihood ratio test showed that inclusion of non-peer reviewed publications did not significantly change the results (*p* value 0.84). Non-peer reviewed publications were therefore retained in all analyses.

**Figure 2.**
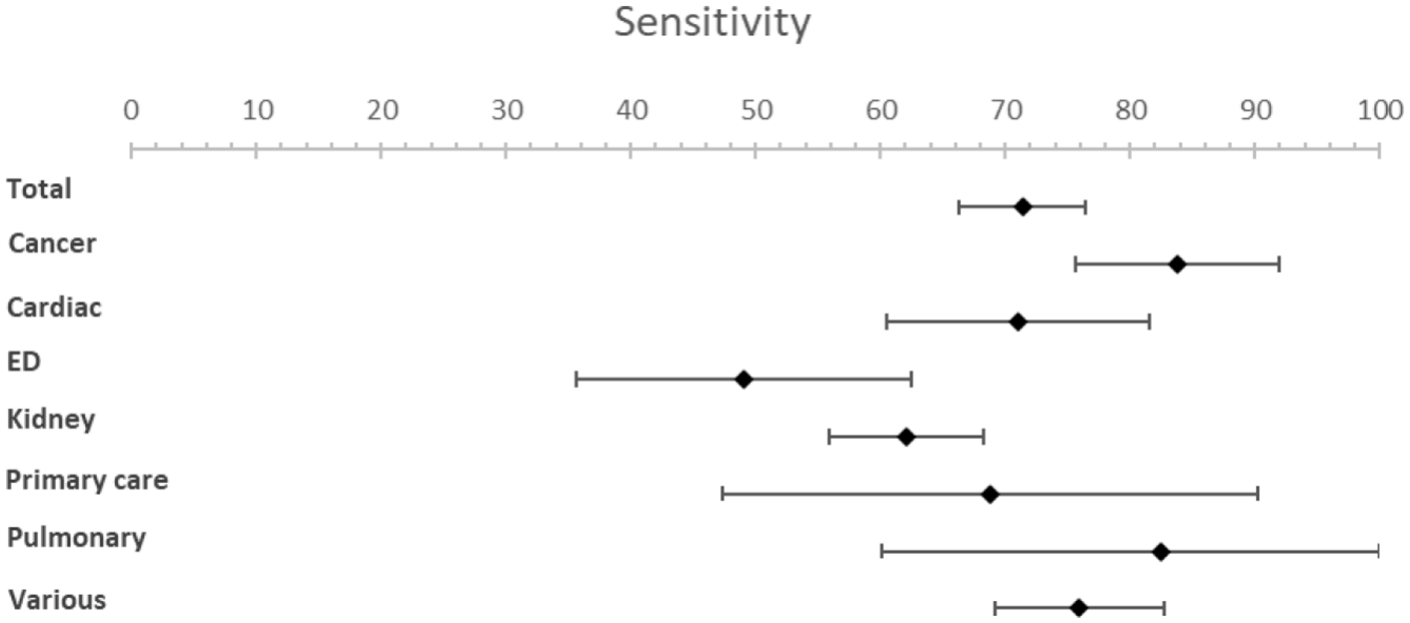
Forest plots for sensitivity.

**Figure 3.**
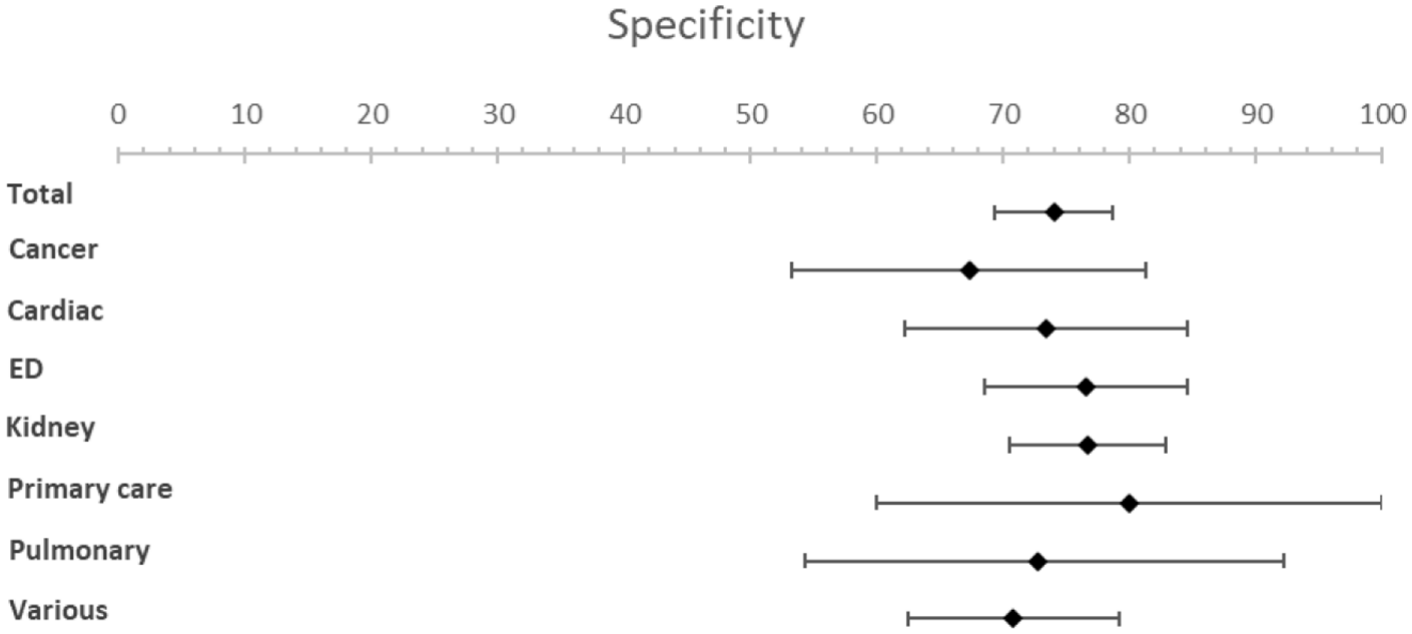
Forest plots for specificity.

**Figure 4.**
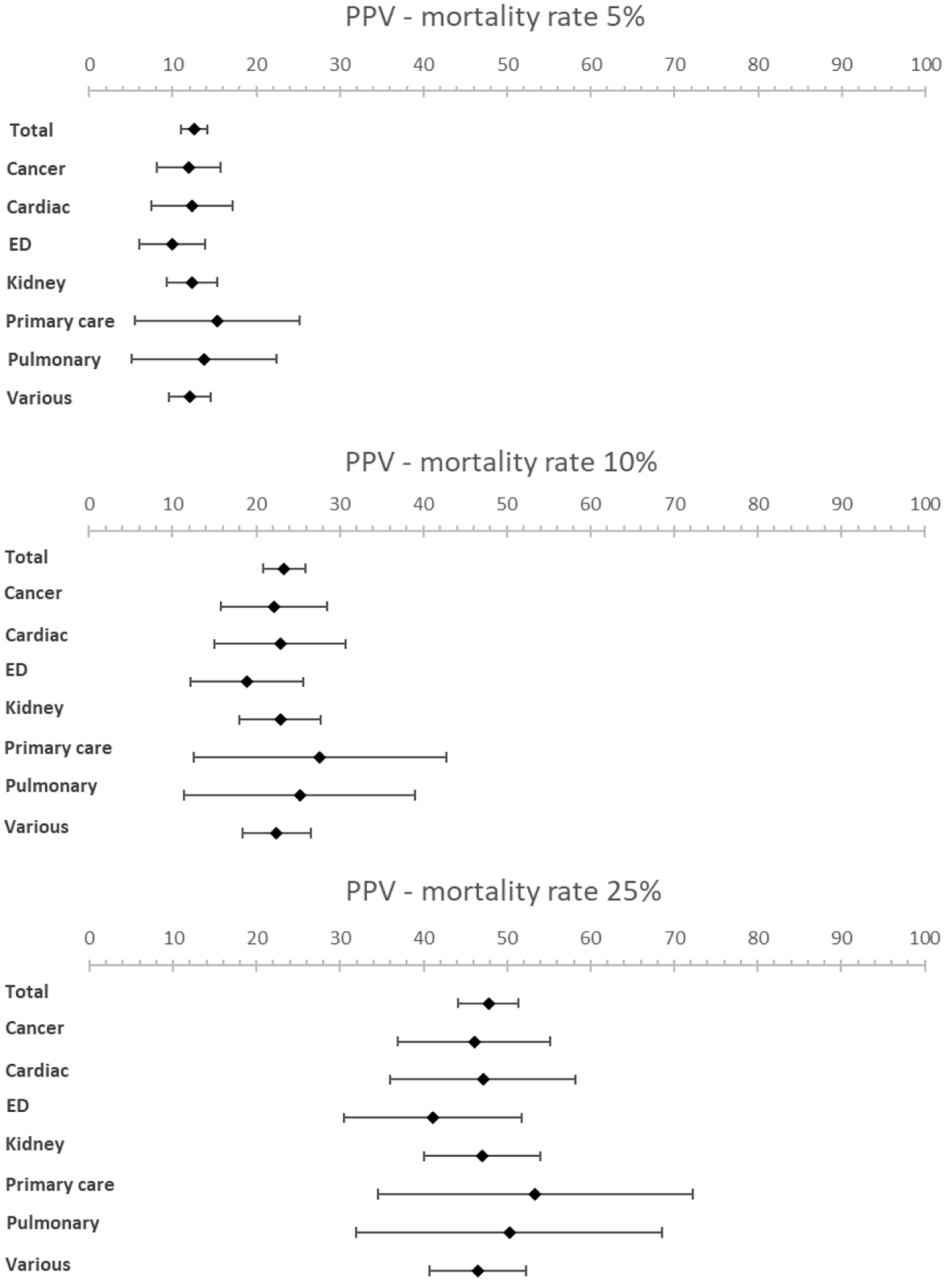
Forest plots showing PPV for various mortality rates (5%, 10% and 25%).

**Figure 5.**
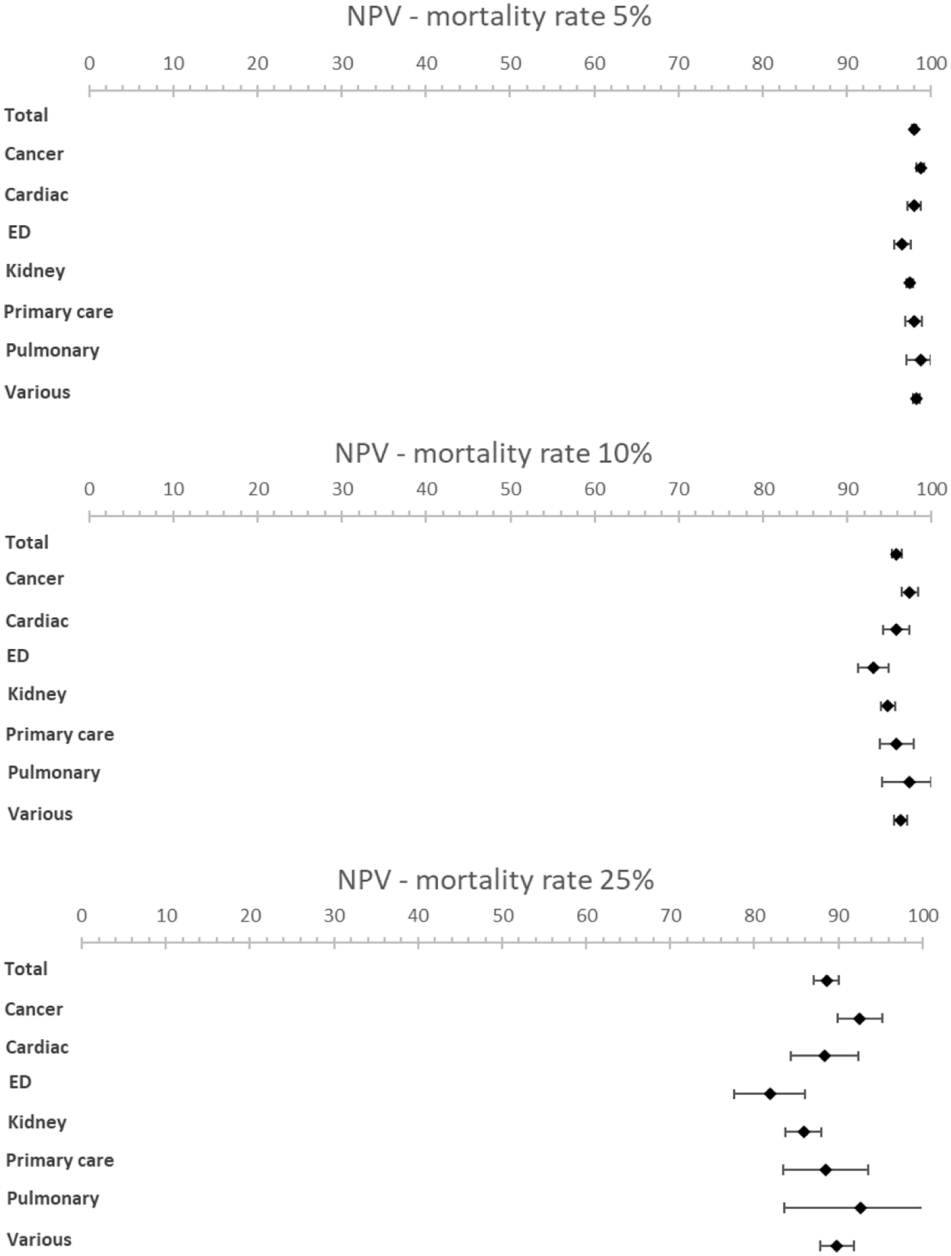
Forest plots showing NPV for various mortality rates (5%, 10% and 25%).

The meta-analysis resulted in an estimated sensitivity of 71.4% (95% CI [66.3–76.4]), an estimated specificity of 74.0% (95% CI [69.3–78.6]) (Table 1, Figures 2 and 3). The estimated NPV varied from 98.0% (95% CI [97.7–98.3]) to 88.6% (95% CI [87.1–90.0]) with a mortality rate of 5% and 25% respectively (Table 1, Figures 4 and 5). The estimated PPV varied from 12.6% (95% CI [11.0–14.2]) with a mortality rate of 5%–47.8% (95% CI [44.2–51.3]) with a mortality rate of 25%. The c-statistic value was 0.79 (95% CI [0.77–0.81]) in the overall analysis. Heterogeneity (*I*^2^) in the overall analysis was 98.2% and 98.4% for sensitivity and specificity respectively.

**Table 1.** Diagnostic accuracy of the surprise question.

Patient subgroup	No. of cohorts	Sensitivity [95% CI]	*I*^2^, %	Specificity [95% CI]	*I*^2^, %	AUC [95% CI]	PPV – mortality rate 5% [95% CI]	NPV – mortality rate 5% [95% CI]	PPV – mortality rate 10% [95% CI]	NPV – mortality rate 10% [95% CI]	PPV – mortality rate 25% [95% CI]	NPV – mortality rate 25% [95% CI]
Total	63	71.4 [66.3–76.4]	98.2	74.0 [69.3–78.6]	98.4	0.79 [0.77–0.81]	12.6 [11.0–14.2]	98.0 [97.7–98.3]	23.4 [20.8–25.9]	95.9 [95.3–96.4]	47.8 [44.2–51.3]	88.6 [87.1–90.0]
Timeframe												
6 months	7	74.5 [67.6–81.4]	96.4	64.3 [56.8–71.8]	97.4	0.75 [0.71–0.80]	9.9 [8.3–11.6]	98.0 [97.5–98.4]	18.8 [16.0–21.7]	95.8 [94.8–96.7]	41.0 [36.5–45.5]	88.3 [85.9–90.8]
12 months	48	73.4 [68.2–78.6]	98.0	72.9 [67.6–78.1]	98.3	0.80 [0.77–0.82]	12.5 [10.8–14.2]	98.1 [97.8–98.4]	23.1 [20.4–25.9]	96.1 [95.5–96.7]	47.5 [43.6–51.3]	89.2 [87.7–90.7]
Subgroups												
Cancer^a^	12^a^	83.8 [75.6–92.0]	90.4	67.3 [53.2–81.3]	90.7	0.83 [0.79–0.88]	11.9 [8.0–15.7]	98.8 [98.3–99.2]	22.2 [15.8–28.5]	97.4 [96.4–98.4]	46.1 [37.0–55.1]	92.6 [89.9–95.2]
Cardiac	7	71.0 [60.5–81.5]	82.4	73.4 [62.2–84.6]	86.9	0.78 [0.69–0.87]	12.3 [7.5–17.1]	98.0 [97.2–98.8]	22.9 [15.0–30.8]	95.8 [94.2–97.4]	47.1 [36.0–58.2]	88.4 [84.3–92.4]
ED	5	49.1 [35.7–62.5]	80.9	76.5 [68.5–84.6]	82.8	0.68 [0.58–0.79]	9.9 [6.0–13.8]	96.6 [95.7–97.6]	18.9 [12.2–25.6]	93.1 [91.3–94.9]	41.1 [30.5–51.7]	81.9 [77.7–86.1]
Kidney	18	62.1 [55.9–68.3]	91.4	76.7 [70.5–83.0]	94.4	0.76 [0.70–0.81]	12.3 [9.3–15.3]	97.5 [97.0–97.9]	22.9 [18.0–27.8]	94.8 [93.9–95.7]	47.1 [40.1–54.0]	85.9 [83.7–88.0]
Primary care	7	68.8 [47.3–90.3]	86.8	80.0 [60.0–99.9]	87.5	0.81 [0.74–0.89]	15.3 [5.5–25.1]	98.0 [97.0–99.0]	27.6 [12.5–42.7]	95.8 [93.9–97.8]	53.4 [34.5–72.2]	88.5 [83.4–93.6]
Pulmonary^b^	6^b^	82.5 [60.1–100.0]	79.1	72.8 [54.3–91.3]	82.8	0.85 [0.68–1.00]	13.8 [5.1–22.5]	98.8 [97.1–100.0]	25.2 [11.4–39.0]	97.4 [94.1–100.0]	50.3 [32.0–68.6]	92.6 [83.6–100.0]
Various	12^c^	75.9 [69.2–82.7]	89.9	70.8 [62.5–79.2]	91.3	0.80 [0.76–0.83]	12.1 [9.6–14.5]	98.2 [97.9–98.6]	22.4 [18.4–26.5]	96.4 [95.6–97.1]	46.5 [40.7–52.2]	89.8 [87.8–91.8]
Type of publication												
Peer reviewed	47^d^	72.0 [66.4–77.6]	97.7	73.9 [68.6–79.3]	97.9	0.79 [0.77–0.82]	12.7 [10.9–14.5]	98.1 [97.7–98.4]	23.5 [20.5–26.4]	96.0 [95.3–96.6]	47.9 [43.8–52.1]	88.8 [87.2–90.4]
Non-peer reviewed	16^e^	69.1 [58.4–79.8]	93.0	74.1 [64.9–83.3]	93.9	0.78 [0.73–0.83]	12.3 [9.2–15.4]	97.9 [97.3–98.5]	22.9 [17.9–27.9]	95.6 [94.4–96.8]	47.1 [40.0–54.2]	87.8 [84.8–90.8]

ED: emergency department; AUC: area under the curve; *I*^2^: Heterogeneity; CI: confidence interval; PPV: positive predictive value; NPV: negative predictive value.

a 2/12 cohorts were analysed with two separate timeframes.

b 1/6 cohort was analysed with two separate timeframes.

c 1/12 cohort was analysed with two separate timeframes.

d 3/47 cohorts were analysed with two separate timeframes.

e 1/16 cohort was analysed with two separate timeframes.

Results from the subgroup analysis including timeframe subgroups (6- and 12-months), patient subgroups and peer reviewed versus non-peer reviewed subgroups can be found in Table 1 and Figures 2 to 5. Analysis of timeframe subgroups showed similar sensitivity for 6- and 12-month timeframe: 74.5% (95% CI [67.6–81.4]) and 73.4% (95% CI [68.2–78.6]) respectively. Specificity was lower for a 6-month timeframe 64.3% (95% CI [56.8–71.8]) compared to a 12-month timeframe 72.9% (95% CI [67.6–78.1]).

Subgroup analysis of patient subgroups showed a lower sensitivity for the surprise question at the emergency department (49.1%; 95% CI [35.7–62.5]) compared to higher sensitivities for cancer patients (83.8%; 95% CI [75.6–92.0]) and patients with pulmonary disease (82.5%; 95% CI [60.1–100]). Specificity varied from 67.3% (95% CI [53.2−81.3]) in cancer patients to 80.0% (95% CI [60.0–99.9]) in primary care patients. NPV was the lowest in the emergency department with a NPV of 96.6% (95% CI [95.8–97.6]) and the highest in pulmonary patients with a NPV of 98.8% (95% CI [97.1–100.0]) at a mortality rate of 5%. NPV varied from 81.9% (95% CI [77.7–86.1]) in patients at the emergency department to 92.6% (95% CI [83.6–100]) in patients with pulmonary disease and 92.6% (95% CI [89.9–95.2]) in patients with cancer at a mortality rate of 25%.

In seven studies multiple healthcare professionals answered the surprise question. Due to the heterogeneity of the results (different patient subgroups, different healthcare professionals answering the surprise question with different seniority and different intensity in care provision to the patient) we could not perform a meta-analysis on this subgroup. An overview of the accuracy of the surprise question by different healthcare professionals can be found in Table 2. The study by Da Silva Gane et al.^26^ investigated the variability between nephrologists and nurses of different levels of seniority (referred to as ‘bands’). They conclude that nephrologists perform better compared to nurses based on a higher sensitivity and similar specificity. The study of Lakin et al.^57^ also show that primary care physicians have a higher sensitivity compared to nurse care coordinators. On the contrary, the results of Valerio and Farinha^75^ show that nurses have a higher sensitivity and lower specificity compared to nephrologists and the results of Straw et al.^60^ show that heart failure nurses have a higher sensitivity compared to cardiologists, trainee-grade doctors and non-specialist nurses. Similar performances between healthcare professionals are seen in the study by Mudge et al.^50^ when comparing doctors and senior nurses and by Rauh et al.^71^when comparing doctors, nurses and advanced practice providers. Ebke et al.^45^ compare the accuracy of answering the surprise question by neurorehabilitation physicians and palliative care physicians, with palliative care physicians having a higher sensitivity and lower specificity. In five other studies multiple healthcare professionals answered the surprise question, however, no separate data was reported.^40,52,55,68,74^

**Table 2.** Accuracy of the surprise question by type of healthcare professional.

Study	Type of healthcare professional	Sensitivity, % [95% CI]**	Specificity, % [95% CI]**	PPV, % [95% CI]**	NPV, % [95% CI]**
Da Silva Gane et al.^26^	Nephrologists	73.7 [64.6–82.8]	73.9 [66.3–81.5]	33.5 [27.3–39.7]	94.3 [90.9–97.7]
	Nurse band 5*	35.6 [21.3–49.9]	85.4 [77.8–93.0]	32.4 [21.7–43.1	88.3 [86.5–90.1]
	Nurse band 6*	51.1 [31.5–70.7]	78.5 [69.1–87.9]	31.1 [24.4–37.8]	90.1 [87.3–92.9]
	Nurse band 7/8*	51.4 [18.1–33.1]	79.1 [72.4–85.8]	30.0 [20.7–39.3]	90.3 [87.8–92.8]
Ebke et al.^45^	Neurorehabilitation physicians	50.0 [32–67]	86.1 [81–91]	37.8 [27–50]	91.1 [88–94]
	Palliative care physicians	67.7 [50–83]	70.3 [64–77]	27.7 [22–34]	92.8 [89–96]
	At least one clinician	76.5	64.9	26.8	94.2
Mudge et al.^50^	Doctors	81	70	38	94
	Senior nurses	80	68	36	93
	Either discipline	90	56	31	96
Straw et al.^60^	Cardiologists	85	59	52	88
	Trainee-grade doctor	75	62	51	83
	Heart failure nurse	90	44	45	90
	Non-specialist nurse	66	73	58	79
Lakin et al.^57^	Primary care physician	79.4	68.6	31.6	94.8
	Nurse care coordinators	52.6	80.6	31.8	90.8
	Either healthcare professional says ‘no’	82.6	62.7	28.1	95.3
	Both healthcare professionals say ‘no’	50.3	86.7	40	90.8
Valerio and Farinha^75^	Nephrologists	68.2	77.3	27.8	95
	Nurses	81.8	64.5	22.8	96.5
Rauh et al.^71^	Medical doctor	75	69	43	90
	Nurses	71	61	42	84
	Advanced practice providers	83	67	44	93
	Combined	75	66	43	89

PPV: positive predictive value; NPV: negative predictive value.

* Band 5 nurses are less senior nurses. Band 6 nurses are of intermediate seniority and band 7/8 are senior nurses.

** CI’s are only provided when presented in the original study.

## Discussion

### Main findings

This meta-analysis evaluated the accuracy of the surprise question in predicting death, differentiating by timeframe, patient subgroup and by type of healthcare professional answering the surprise question. In total, 59 studies encompassing 63 cohorts were identified including 88.268 surprise question assessments. The pooled sensitivity was 71.4% (95% CI [66.3–76.4]) and the pooled specificity 74.0% (95% CI [69.3–78.6]). The c-statistic value was 0.79 (95% CI [0.77–0.81]) in the overall analysis. Analysis of timeframe subgroups showed similar sensitivity for 6- and 12-month timeframe (74.5% (95% CI [67.6–81.4]) and 73.4% (95% CI [68.2–78.6]) respectively) and lower specificity for 6-month timeframe compared to a 12-month timeframe (64.3% (95% CI [56.8–71.8]) and 72.9% (95% CI [67.6–78.1]) respectively). Pooled estimates showed variation between patient groups. A sensitivity of 83.8% (95% CI [75.6–92.0]) was observed for patients with cancer and 82.5% (95% CI [60.1–100]) for patients with pulmonary disease, whereas the sensitivity for the emergency department was 49.1 (95% CI [35.7–62.5]). Specificity showed less variation with values between 67.3% (95% CI [53.2 and 81.3]) for cancer patients and 80.0% (95% CI [60.0–99.9]) for primary care patients. The estimated NPV varied from 98.0% (95% CI [97.7–98.3]) to 88.6% (95% CI [87.1–90.0]) with a mortality rate of 5% and 25% respectively. The estimated PPV varied from 12.6% (95% CI [11.0 to 14.2]) with a mortality rate of 5% to 47.8% (95% CI [44.2–51.3]) with a mortality rate of 25%. The NPV remains high with increasing mortality rate in all subgroups. Seven studies provided detailed information on different healthcare professionals answering the surprise question. Based on these studies we did not find clear evidence for a difference between the accuracy of healthcare professionals answering the surprise question.

### Strengths and limitations

This study has a number of strengths. First of all, each part of the review process was independently undertaken by two reviewers. Furthermore, a high number of studies have been included. This can be explained by (1) the increased attention for palliative care and the surprise question, resulting in a high amount of recently published studies (2) the effort made to obtain additional data by contacting authors and (3) including non-peer reviewed studies: 16 of the 59 included studies were non-peer reviewed studies, mostly conference abstracts. We also included the non-peer reviewed studies in an effort to avoid publication bias of favourable outcomes.^104^ A limitation of including non-peer reviewed studies is that they did not provide sufficient information for a comprehensive quality assessment, which could have led to a relatively negative quality assessment. Furthermore, we observed a high degree of heterogeneity, with an overall *I*^2^ of 98.2% and 98.4% for sensitivity and specificity respectively. The analysis with subgroups (i.e. timeframe, patient subgroups and type of publication) still showed a high degree of heterogeneity. This can be explained by the enormous diversity in included studies, reflecting the different real-life circumstances in which the surprise question is used, and its versatile nature. Furthermore, the accuracy of the surprise question may be overestimated due to a possible self-fulling prophecy: a positive answer to the surprise question (‘No, I would not be surprised’) could lead to, consciously or subconsciously, discussing goals of care, thereby potentially influencing outcome. Finally, c-statistics were estimated with an easy to apply formula, which may result in a slight over-estimation.^13^

### Comparison to other literature

As described earlier, two meta-analyses were performed on the accuracy of the surprise question by Downar et al.^3^ and White et al.^4^ Despite this, the subjectiveness and accuracy of using the surprise question are still debated.^105,106^ The previous meta-analyses included 17 and 22 cohorts, with 11.621 and 25.718 surprise question assessments respectively, compared to 63 cohorts and 88.268 SQ assessments in this study. Moreover, Downar et al. did not include ‘Gold Standards Framework’ in the search, therefore missing studies that did not mention the surprise question in title or abstract. Furthermore, both meta-analyses report a substantial risk of bias in their included studies. Indeed, in our assessment, most pre-2017 studies have an increased risk of bias whereas more recent studies seem to be of better methodological quality. Hence, our results may be more reliable due to the increase of surprise question assessments included and improved methodological quality of included studies.

This study shows similar results in overall accuracy in predicting death compared to the previous meta-analyses. Downar et al. reported a sensitivity of 67.0% and a specificity of 80.2% compared to 71.4% and 74.0% respectively in our study. The c-statistic (area under the curve) of Downar et al.^3^ was 0.81 [0.78–0.84] compared to 0.79 [0.77–0.81] in our meta-analysis. De Bock et al.^107^ studied the accuracy of the Supportive and Palliative Care Indicators Tool (SPICT) in a geriatric population and report a higher sensitivity of 84.1% and a lower specificity of 57.9% compared to our results of the surprise question.

White et al. stated that an increase in timeframe did not impact the diagnostic accuracy. Our study showed similar sensitivity for 6- and 12-month timeframe. However we found a lower specificity for 6-month timeframe compared to a 12-month timeframe. Our study confirms the previous conclusions that the surprise question performs better in cancer patients compared to other subgroups. We did not find clear evidence for a difference between the accuracy of healthcare professionals answering the surprise question, in contrast to an earlier suggestion by White et al.^4^ that doctors seem to be more accurate than nurses in recognising people in the last year of life.

### Implications for practice

A systematic review by Cardona-Morrell et al.^108^ indicated that on average 33%–38% of patients nearing their end of life receive non-beneficial treatments in the last 6 months of their life. Advance care planning can have a positive effect on end of life care, decrease life-sustaining treatment, increase use of hospice and palliative care, prevent hospital admissions and improve goal-concordant care.^109^ Timely identification of patients who could potentially benefit from advance care planning is important.^110^ The importance of advance care planning increases when nearing the end of life. Hence, prognostication of mortality can be used as a proxy for initiating advance care planning. The surprise question is an easy to use tool^2^ and does not require large amounts of clinical data compared to other available screening tools.^111^ These characteristics and the reasonable accuracy in predicting death with fairly high NPV with various mortality rates make the surprise question an appropriate screening tool for initiating advance care planning. Additionally, patients with a positive answer to the surprise question (‘No, I would not be surprised’) are likely to be vulnerable and may therefore benefit from advance care planning regardless of whether they die exactly within the specified timeframe. Furthermore, initiating advance care planning ‘too early’ does not seem to cause damage.^109^ The results of this systematic review and meta-analysis encourage the use of the surprise question as screening tool by various healthcare professionals, not exclusively by doctors. We think the surprise question should not solely be seen as an indicator of prognostication of death but rather as an opportunity for renewed attention for quality of care and shared decision making by timely initiating advance care planning.

## Conclusion

We found overall reasonable test characteristics for the surprise question. Additionally, this study showed notable differences in performance within patient subgroups. However, we did not find an indication of notable differences between timeframe and healthcare professionals. We submit that the surprise question is an appropriate tool for initiating advance care planning.

## Figures and Tables

**Appendix 5.**
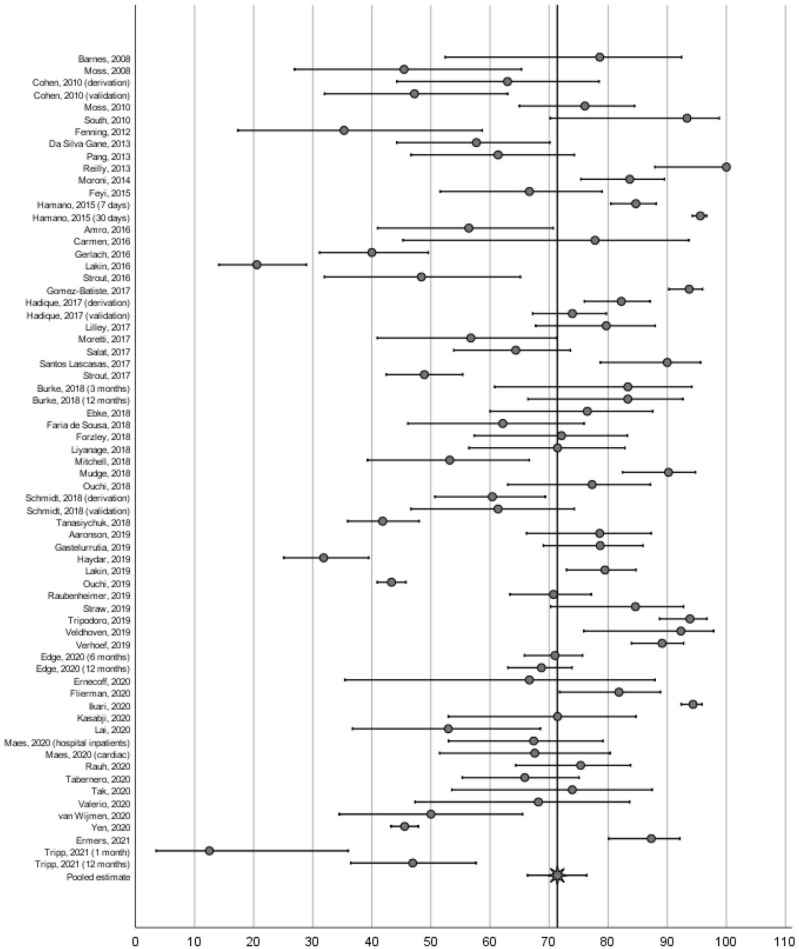
Forest plot of the sensitivity of individual studies.

**Appendix 6.**
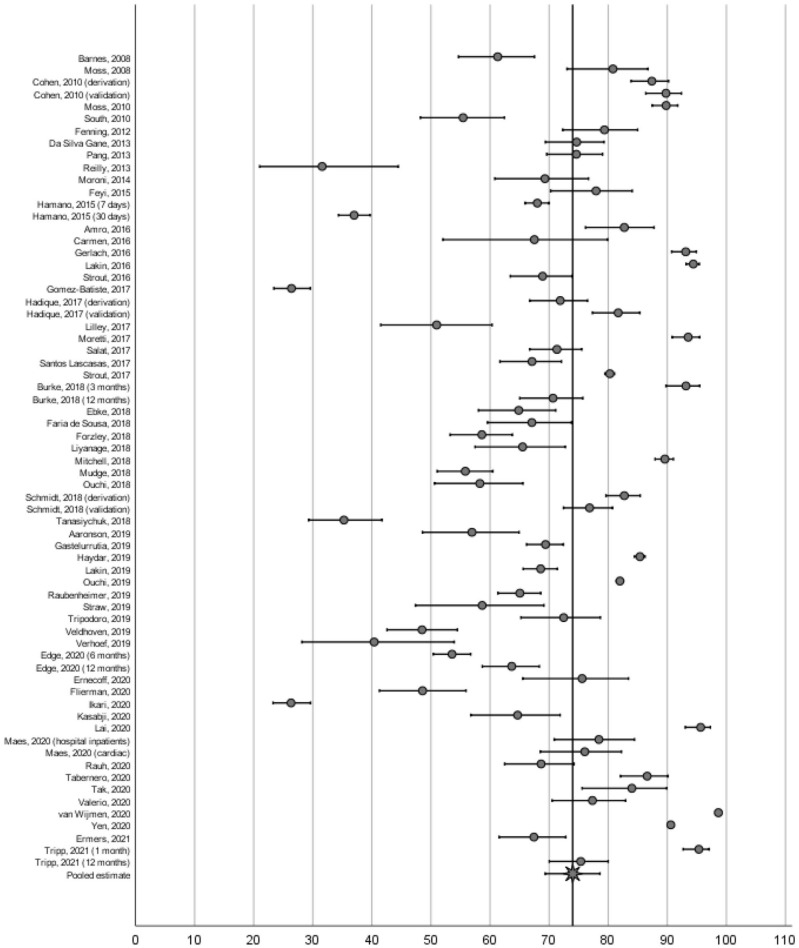
Forest plot of the specificity of individual studies.

## Appendix

**Appendix 1.** Search strategy.

Source	Date of search	Search strategy
PubMed	22-01-2021	((surprise question*[Title/Abstract]) OR “gold standards framework”[Title/Abstract]) OR NECPAL[Title/Abstract]
Embase	22-01-2021	‘surprise question*’:ti,ab,kw OR ‘gold standards framework’:ti,ab,kw OR necpal:ti,ab,kw
Cochrane	22-01-2021	(‘Gold Standards Framework’):ti,ab,kw OR (‘surprise question’):ti,ab,kw OR (NECPAL):ti,ab,kw
Scopus	22-01-2021	TITLE-ABS-KEY (‘surprise question*’) OR TITLE-ABS-KEY ( ‘gold standards framework’ ) OR TITLE-ABS-KEY
Web of Science	22-01-2021	TOPIC:(‘surprise question*’) OR TOPIC:(‘gold standards framework’) OR TOPIC: (NECPAL)
CINAHL	22-01-2021	TI ‘surprise question*’ OR AB ‘surprise question*’ OR MH ‘surprise question*’ OR TI ‘gold standards framework’ OR AB ‘gold standards framework*’ OR MH ‘gold standards framework*’ OR TI ‘necpal*’ OR AB ‘necpal*’ OR MH ‘necpal*’

No filters/limits were applied in the searches.

**Appendix 2.** Characteristics of included studies.

Author	Country	Population	Healthcare professional answering the surprise question	Setting	Total subjects (*n*)	Total surprise question assessments (*n*)	Surprise question timeframe	Subject age mean (SD)	Gender M:F
Barnes et al.^18^	United Kingdom	Heart failure, 60 years or older	General practitioners	General practice	542	231	12 months	77 (71–82)**	121:110****
Moss et al.^21^	United States	Haemodialysis	Nurse practitioners	3 haemodialysis units	147	147	12 months	66.4 (14.8)	81:66
Cohen et al.^22^	United States	Haemodialysis	Nephrologists	Haemodialysis unit	512 (d)	447 (d)	6 months	60 (17) (d)	254:195 (d)
					514 (v)	427 (v)		63 (16) (v)	245:182 (v)
Moss et al.^23^	United States	Breast, lung or colon cancer	Oncologists	Outpatient clinic	853	826	12 months	60 (13)	126:727
South et al.^24^ (a)	United Kingdom	Acute exacerbation COPD	NR	COPD unit	199	199	6–12 months	70 (NR)	92:107
Fenning et al.^25^	United Kingdom	Acute coronary artery disease	Medical staff	Cardiology ward	172	172	6–12 months	66 (14)	105:67
Da Silva Gane et al.^26^	United Kingdom	Haemodialysis	Nephrologists and nurses	3 haemodialysis units	344	344	12 months	71.9 (11.0) (died) 62.2 (15.8) (alive)	221:123
Pang et al.^27^	Hong Kong	Peritoneal dialysis patients	Nephrologists	Dialysis centre	367	367	12 months	60.2 (12.3)	205:162
Reilly et al.^28^ (a)	United Kingdom	Non-cancer respiratory patients	Pulmonologists	Ward of general hospital	85	85	12 months	68 (NR)	36:49
Moroni et al.^29^	Italy	Stage IV cancer	General practitioners	General practice	231	231	12 months	70.2 (SE 0.9)	117:114
Feyi et al.^30^	United Kingdom	End-stage renal disease	Multidisciplinary	1 dialysis unit	178	178	12 months	NR	NR
Hamano et al.^31^	Japan	Advanced cancer	Palliative care physicians	58 palliative care services	2361	2361	7 and 30 days	69.1 (12.8)	1358:1003
Amro et al.^32^	United States	Haemodialysis	Nephrologists	Dialysis centre	201	201	12 months	71 (65–77)**	105:96
Maria Carmen et al.^33^ (a)	Spain	Haemodialysis	Medical staff	Haemodialysis unit	49	49	12 months	NR	NR
Gerlach et al.^34^ (a)	Germany	Cancer (haemato-oncology)	Physicians	Hemato-oncology outpatient clinic	828	672	12 months	NR	NR
				Stem cell transplantation unit					
Lakin et al.^35^ (a)	United States	High risk patients	General practitioners	Academic primary care centre	1737	1737	12 months	65 (NR)	696:1041
Strout et al.^36^ (a)	United States	Emergency department patients with sepsis	Emergency physicians	Emergency department	330	330	30 days	71 (0–97)***	182:148
Gómez-Batiste et al.^37^	Spain	Advanced chronic disease	Healthcare professional	Three primary care centres, one general hospital, one intermediate care centre, four nursing homes	1064	1059	12 months	81.3 (11.8)	378:686
Hadique et al.^38^	United States	Medical ICU patients	ICU physicians	Medical ICU	500 (d)	500 (d)	6 months	61.1 (17.7) (d)	257:243
					549 (v)	543 (v)		61.1 (16.2) (v)	296:253
Lilley et al.^39^	United States	Acute surgical conditions, 65 years or older	Surgical residents and attendings	Emergency general surgery service	119	163*	12 months	79.3 (7.9) (SQ+)	78:85
								73.5 (7.0) (SQ−)	
Moretti et al.^40^	United Kingdom; Italy	Acute coronary artery disease	Medical and nursing staff	Cardiology ward	629	470	6–12 months	67.5 (12.7)	432:197
Salat et al.^41^	United States	Advanced chronic kidney disease (no dialysis) >60 years	Nephrologists	Outpatient clinic	488	488	12 months	71 (65–77)*	239:249
Santos Lascasas et al.^42^ (a)	Portugal	Dialysis patients, 65 years or older	NR	Dialysis unit	360	360	6 months	76 (NR)	169:191
Strout et al.^43^ (a)	United States	Admitted adult emergency department patients	Emergency physician and inpatient physician	Emergency department	10,334	9923	30 days	67 (18–103)***	50.3% female
				Hospital wards					
Burke et al.^44^	United Kingdom	Children (0–21 years) with life-threatening disease	Multidisciplinary team	Children’s hospice	327	325 (3 m) 306 (12 m)	3 months and 12 months	7.7 (5.3)	185:142
Ebke et al.^45^	Germany	Neurorehabilitation	Neurologists and palliative care physicians	Neurorehabilitation centre	279	236	12 months	63 (14)	161:118
Faria de Sousa et al.^46^ (a)	Portugal	Advanced stages of: cancer, heart failure, kidney disease, COPD	General practitioners	General practice	209	201	6 months	72.6 (12.6)	113:96
Forzley et al.^47^	Canada	Haemodialysis	Nephrologists	Haemodialysis unit	374	374	6 months	68 (15)	221:153
Liyanage et al.^48^	Australia	Nursing home residents	Director of Nursings	Nursing home	187	187	12 months	82.4 (9.09)	83:104
Mitchell et al.^49^	Australia	Patients, 70 years or older	General practitioners	19 general practices	4375	1525	12 months	79.1 (6.9)**	1013:512****
Mudge et al.^50^	Australia	Hospital inpatients	Senior nurses and medical staff	Teaching hospital	524	513	12 months	60.2 (18.9)	276:237****
Ouchi et al.^51^	United States	Emergency department patients, 65 years or older	Emergency physicians	Emergency department	207	207	12 months	75 (7.5)	NR
Schmidt et al.^52^	United States	Advanced chronic kidney disease (no dialysis)	Clinicians and nurse practitioners	Outpatient clinics	749 (d)	749 (d) 437 (v)	12 months	69.3 (14.6) (d) 70.0 (13.3) (v)	381:368 (d)166:271 (v)
					437 (v)				
Tanasiychuk et al.^53^	Israel	Acute Kidney Injury with unscheduled dialysis	Nephrologists	Hospital	475	475	6 months	72.8 (12.2)	238:192
Aaronson et al.^54^	United States	Heart failure	Emergency physicians	Emergency department	193	193	12 months	74.5 (12.6)	NR
Gastelurrutia et al.^55^ (a)	Spain	Heart failure	Physicians and nurses	3 outpatient heart failure clinics	922	922	12 months	69.3 (12.2)	648:274
Haydar et al.^56^	United States	Emergency department patients, 18 years or older	Emergency physicians	Emergency department	6122	6089	30 days	66 (51–79)**	3168:2954
Lakin et al.^57^	United States	Chronically ill primary care patients	Primary care physician and nurse care coordinators	Primary care	1163	1163*	24 months	70.1 (14.8)	500:663
Ouchi et al.^58^	United States	Admitted Emergency department patients, 65 years or older	Attending physicians	Emergency department	10,737	16,223*	30 days	75.9 (8.8)	5205:5532
Raubenheimer et al.^59^	South Africa	Admitted to acute medical services	Two trained clinicians	Acute medical services	822	822	12 months	52 (37–67)**	378:444
Straw et al.^60^	United Kingdom	Decompensated heart failure	Cardiologists. Trainee-grade doctors and heart failure nurses	Cardiology ward	129	114	12 months	71 (14)	73:41
Tripodoro et al.^61^	Argentina	Cancer	Treating physicians	All patients in 1 centre	317	313	12 months	77 (21–99)***	106:211
Veldhoven et al.^62^	The Netherlands	Primary care patients, 75 years or older	Two general practitioners	General practice	292	292	12 months	84 (5.46)	117:175
Verhoef et al.^63^	The Netherlands	Advanced cancer at emergency department	Attending physicians	Emergency department	245	245	12 months	62 (45–79)**	118:127
Edge et al.^64^ (a)	United States	Metastatic cancer	Oncologists	Outpatient clinic	1276 (6 m) 655 (12 m)	1276 (6 m) 655 (12 m)	6 and 12 months	NR	NR
Ernecoff et al.^65^ (a)	United States	Advance chronic kidney patients, 60 years or older	Physicians	Outpatient nephrology clinic	510	95	12 months	NR	NR
Flierman et al.^66^	The Netherlands	Hospital inpatients, 70 years or older	Nurses	Ward of general hospital	252	252	12 months	81.2 (6.56)	122:130
Ikari et al.^67^	Japan	Metastatic cancer	Physicians	Palliative care unit	1411	1411	3 days	72.6 (12.2)	716:659
Estifan Kasabji et al.^68^ (a)	Spain	Haemodialysis	Nephrologists and nurses	Haemodialysis unit	180	178	6–12 months	69 (14.1)	124:56
Lai et al.^69^	Taiwan	Peritoneal dialysis patients	Nurses	Haemodialysis unit	401	401	12 months	56.2 (14)	214:213
Maes et al.^70^	Belgium	Hospital inpatients, 75 years or older: first cohort acute geriatric ward; second cohort cardiac patients	Physicians	Geriatric ward and cardiology ward	458	368	6–12 months	NR	184:195
Rauh et al.^71^	United States	Gynaecological oncology	Attending physicians, ‘physician assistants, nurses’	Two tertiary centres	358	341	12 months	NR	0:358
Tabernero Huguet et al.^72^ (a)	Spain	Patients on respiratory ward	NR	Respiratory ward of hospital	363	361	12 months	NR	NR
Tak et al.^73^ (a)	The Netherlands	Idiopathic pulmonary fibrosis	Health care providers	Outpatient clinic	123	123	12 months	74 (63)	108:15
Valerio and Farinha^75^ (a)	Portugal	Haemodialysis	Nephrologists and nurses	Haemodialysis unit	194	194	12 months	69.9	NR
van Wijmen et al.^76^	The Netherlands	General population	General practitioners	General practice	3640	3640	12 months	NR	NR
Yen et al.^77^	Taiwan	Hospital inpatients	Nurses	Hospital	23,444	21,098	6–12 months	62.8	11,230:9879
Ermers et al.^78^	The Netherlands	Cancer	Oncologists	Outpatient clinic	382	379	12 months	59.4	211:168
Tripp et al.^74^	United States	COPD patients	Physicians and advanced practice providers	Emergency department (30 days) and general ward of hospital (12 months)	428	381 (30 days) 365 (12 months)	30 days and 12 months	NR	210:218

(a): abstract or other non-peer reviewed publication; NR: not reported; (d): derivation cohort; (v): validation cohort; SQ+: surprise question answered with ‘no’; SQ−: surprise question answered with ‘yes’.

* Multiple assessments per patient possible. **Median (IQR). ***Median (range). ****SQ group only.

**Appendix 3.** Critical appraisal.

Article	1. Study participation	2. Study attrition	3. Prognostic factor measurement	4. Outcome measurement	5. Study confounding	6. Statistical analysis and reporting	Total
Barnes et al.^18^	High	Low	Low	Low	Low	Low	Low
Moss et al.^21^	Low	Low	Low	Low	Moderate	Low	Low
Cohen et al.^22^	Moderate	Low	Low	Low	Moderate	Low	Low
Moss et al.^23^	Low	Low	Low	Low	Low	Low	Low
South et al.^24^ (a)	High	Low	Moderate	High	Moderate	Low	High
Fenning et al.^25^	Moderate	Low	Low	Low	Low	Low	Low
Da Silva Gane et al.^26^	Low	Low	Low	Low	Moderate	Low	Low
Pang et al.^27^	Low	Low	Low	Low	Low	Low	Low
Reilly et al.^28^ (a)	High	Low	Moderate	Moderate	Moderate	Low	Moderate
Moroni et al.^29^	Moderate	Low	Low	Low	Low	Low	Low
Feyi et al.^30^	High	Low	High	High	High	Low	High
Hamano et al.^31^	Moderate	Moderate	Low	Low	Low	Low	Low
Amro et al.^32^	Low	Low	Low	Low	High	Low	Moderate
Maria Carmen et al.^33^ (a)	Moderate	Low	High	Low	Moderate	Low	Moderate
Gerlach et al.^34^ (a)	Moderate	Low	Moderate	Moderate	Low	Low	Moderate
Lakin et al.^35^ (a)	High	Low	Moderate	Low	Low	Low	Low
Strout et al.^36^ (a)	Moderate	Low	Low	Moderate	Low	Low	Moderate
Gómez-Batiste et al.^37^	Low	Low	Low	Low	Low	Low	Low
Hadique et al.^38^	Low	Low	Low	Low	Low	Low	Low
Lilley et al.^39^	Moderate	Low	Low	Low	Low	Low	Low
Moretti et al.^40^	Moderate	Low	Low	Moderate	Low	Low	Low
Salat et al.^41^	Moderate	Low	Low	Low	Low	Low	Low
Santos Lascasas et al.^42^ (a)	Moderate	Low	Low	Low	Moderate	Low	Low
Strout et al.^43^ (a)	Moderate	High	Low	Moderate	Low	Low	High
Burke et al.^44^	Moderate	Low	Low	Low	Low	Low	Low
Ebke et al.^45^	Low	High	Low	Low	Low	Low	Moderate
Faria de Sousa et al.^46^ (a)	Moderate	Low	Low	Low	Low	Low	Low
Forzley et al.^47^	Low	Low	Low	Low	Moderate	Low	Low
Liyanage et al.^48^	Low	Low	Low	Low	Low	Low	Low
Mitchell et al.^49^	Moderate	High	Moderate	Low	Low	Low	Moderate
Mudge et al.^50^	Low	Low	Low	Low	Low	Low	Low
Ouchi et al.^51^	Moderate	Low	Low	Low	Low	Low	Low
Schmidt et al.^52^	Moderate	Low	Low	Low	Moderate	Low	Low
Tanasiychuk et al.^53^	Moderate	Low	Low	Low	Moderate	Low	Moderate
Aaronson et al.^54^	Moderate	Low	Low	Low	Low	Low	Low
Gastelurrutia et al.^55^ (a)	Low	Low	Low	Low	Low	Low	Low
Haydar et al.^56^	Low	Low	Low	Low	Low	Low	Low
Lakin et al.^57^	Moderate	Low	Low	Low	Low	Low	Low
Ouchi et al.^58^	Low	Low	Low	Low	Low	Low	Low
Raubenheimer et al.^59^	Moderate	Low	Low	Low	Low	Low	Low
Straw et al.^60^	Moderate	Low	Low	Low	Low	Low	Low
Tripodoro et al.^61^	Low	Low	Low	Low	Low	Low	Low
Veldhoven et al.^62^	Low	Low	Low	Low	Low	Low	Low
Verhoef et al.^63^	Moderate	Low	Low	Low	Low	Low	Low
Edge et al.^64^ (a)	Moderate	Low	Low	Low	Low	Low	Low
Ernecoff et al.^65^ (a)	High	Low	Low	Low	Low	Low	Moderate
Flierman et al.^66^	Moderate	Low	Low	Low	Low	Low	Low
Ikari et al.^67^	Moderate	Low	Low	Low	Low	Low	Low
Estifan Kasabji et al.^68^ (a)	High	Low	Moderate	Low	Moderate	Low	Moderate
Lai et al.^69^	Low	Low	Low	Low	Moderate	Low	Low
Maes et al.^70^	High	Moderate	Low	Moderate	Low	Low	Moderate
Rauh et al.^71^	Moderate	Low	Low	Low	Low	Low	Low
Tabernero Huguet et al.^72^ (a)	Moderate	Low	Low	Low	Moderate	Low	Moderate
Tak et al.^73^ (a)	Moderate	Low	Low	Low	Low	Low	Low
Valerio and Farinha^75^ (a)	Moderate	Low	Low	Low	Moderate	Low	Moderate
Van Wijmen etal.^76^	Low	Low	Low	Low	Low	Low	Low
Yen et al.^77^	Low	Low	Low	Low	Moderate	Low	Low
Ermers et al.^78^	Low	Low	Low	Low	Moderate	Low	Low
Tripp et al.^74^	Low	Low	Low	Low	Moderate	Low	Low

*Note:* (a): abstract or other non-peer reviewed publication.

**Appendix 4.** Individual study results.

Study (authors)	Patient group	Timeframe (months)	Abstract (Yes/No)	Total SQ responses (n)	Mortality rate (%)	Sensitivity (95% CI)*	Specificity (95% CI)*
Barnes et al.^18^	Cardiac disease	12	No	231	6.1	78.6 [52.4–92.4]	61.3 [54.7–67.5]
Moss et al.^21^	Kidney disease	12	No	147	15.0	45.5 [26.9–65.3]	80.8 [73.0–86.7]
Cohen et al.^22^ (derivation cohort)	Kidney disease	6	No	447	6.0	63.0 [44.2–78.5]	87.4 [83.9–90.2]
Cohen et al.^22^ (validation cohort)	Kidney disease	6	No	427	8.4	47.2 [32.0–63.0]	89.8 [86.4–92.4]
Moss et al.^23^	Cancer	12	No	826	8.6	76.1 [65.0–84.5]	89.8 [87.4–91.8]
South et al.^24^	Pulmonary disease	12	Yes	199	7.5	93.3 [70.2–98.8]	55.4 [48.2–62.4]
Fenning et al.^25^	Cardiac disease	12	No	172	9.9	35.3 [17.3–58.7]	79.4 [72.3–85.0]
Da Silva Gane et al.^26^	Kidney disease	12	No	344	15.1	57.7 [44.2–70.1]	74.7 [69.4–79.3]
Pang et al.^27^	Kidney disease	12	No	367	12.0	61.4 [46.6–74.3]	74.6 [69.6–79.1]
Reilly et al.^28^	Pulmonary disease	12	Yes	85	32.9	100 [87.9–100]	31.6 [21.0–44.5]
Moroni et al.^29^	Cancer	12	No	231	45.0	83.7 [75.4–89.5]	69.3 [60.8–76.6]
Feyi et al.^30^	Kidney disease	12	No	178	23.6	66.7 [51.6–79.0]	77.9 [70.3–84.1]
Hamano et al.^31^ – 7 day timeframe	Cancer	0.25	No	2361	14.1	84.7 [80.4–88.2]	68.0 [65.9–70.0]
Hamano et al.^31^ – 1 month timeframe	Cancer	1	No	2361	47.2	95.6 [94.2–96.7]	37.0 [34.4–39.7]
Amro et al.^32^	Kidney disease	12	No	201	19.4	56.4 [41–70.7]	82.7 [76.2–87.8]
Maria Carmen et al.^33^	Kidney disease	12	Yes	49	18.4	77.8 [45.3–93.7]	67.5 [52.0–79.9]
Gerlach et al.^34^	Cancer	12	Yes	672	15.6	40.0 [31.1–49.6]	93.1 [90.7–94.9]
Lakin et al.^35^	Primary care	12	Yes	1737	6.4	20.5 [14.1–28.9]	94.4 [93.2–95.4]
Strout et al.^36^	Emergency department	1	Yes	330	9.4	48.4 [32.0–65.2]	68.9 [63.4–73.9]
Gomez-Batiste, 2017^37^	Primary care	12	No	1059	27.0	93.7 [90.3–96.0]	26.4 [23.4–29.6]
Hadique et al.^38^ (derivation cohort)	Intensive care	6	No	500	36.0	82.2 [76.0–87.1]	71.9 [66.7–76.5]
Hadique et al.^38^ (validation cohort)	Intensive care	6	No	543	34.6	73.9 [67.2–79.7]	81.7 [77.3–85.4]
Lilley et al.^39^	Acute surgical conditions	12	No	163	36.2	79.7 [67.7–88.0]	51.0 [41.5–60.4]
Moretti et al.^40^	Cardiac disease	12	No	470	7.9	56.8 [40.9–71.3]	93.5 [90.8–95.5]
Salat et al.^41^	Kidney disease	12	No	488	17.8	64.4 [53.9–73.6]	71.3 [66.7–75.5]
Santos Lascasas et al.^42^	Kidney disease	6	Yes	360	13.9	90.0 [78.6–95.7]	67.1 [61.7–72.1]
Strout et al.^43^	Emergency department	1	Yes	9923	2.3	48.9 [42.4–55.4]	80.3 [79.5–81.1]
Burke et al.^44^ – 3 month timeframe	Children	3	No	325	5.5	83.3 [60.8–94.2]	93.2 [89.8–95.5]
Burke et al.^44^ – 12 month timeframe	Children	12	No	306	9.8	83.3 [66.4–92.7]	70.7 [65.0–75.7]
Ebke et al.^45^	Neurorehabilitation	12	No	236	14.4	76.5 [60.0–87.6]	64.9 [58.0–71.1]
Faria de Sousa et al.^46^	Primary care	6	Yes	201	18.4	62.2 [46.1–75.9]	67.1 [59.6–73.8]
Forzley et al.^47^	Kidney disease	6	No	374	11.5	72.1 [57.3–83.3]	58.6 [53.2–63.8]
Liyanage et al.^48^	Nursing home	12	No	187	22.5	71.4 [56.4–82.8]	65.5 [57.5–72.8]
Mitchell et al.^49^	Primary care	12	No	1525	3.1	53.2 [39.2–66.7]	89.6 [87.9–91.0]
Mudge et al.^50^	Hospital inpatients	12	No	513	17.9	90.2 [82.4–94.8]	55.8 [51.0–60.5]
Ouchi et al.^51^	Emergency department	12	No	207	21.3	77.3 [63.0–87.2]	58.3 [50.6–65.6]
Schmidt et al.^52^ (derivation cohort)	Kidney disease	12	No	749	13.5	60.4 [50.6–69.4]	82.7 [79.6–85.4]
Schmidt et al.^52^ (validation cohort)	Kidney disease	12	No	437	10.1	61.4 [46.6–74.3]	76.8 [72.4–80.7]
Tanasiychuk et al.^53^	Kidney disease	6	No	475	52.8	41.8 [35.9–48.0]	35.3 [29.3–41.7]
Aaronson et al.^54^	Cardiac disease & emergency department	12	No	193	29.0	78.6 [66.2–87.3]	56.9 [48.6–64.9]
Gastelurrutia et al.^55^	Cardiac disease	12	Yes	922	9.7	78.7 [69.0–85.9]	69.4 [66.2–72.4]
Haydar et al.^56^	Emergency department	1	No	6089	2.6	31.8 [25.1–39.5]	85.4 [84.4–86.2]
Lakin et al.^57^	Primary care	24	No	1163	15.5	79.4 [73.0–84.7]	68.6 [65.6–71.4]
Ouchi et al.^58^	Emergency department	1	No	16,223	9.7	43.3 [40.9–45.8]	82.0 [81.3–82.6]
Raubenheimer et al.^59^	Acute medical services	12	No	822	20.0	70.7 [63.4–77.2]	65.0 [61.3–68.6]
Straw et al.^60^	Cardiac disease	12	No	114	34.2	84.6 [70.3–92.8]	58.7 [47.4–69.1]
Tripodoro et al.^61^	Cancer	12	No	313	46.6	93.8 [88.7–96.7]	72.5 [65.2–78.7]
Veldhoven et al.^62^	Primary care	12	No	292	8.9	92.3 [75.9–97.9]	48.5 [42.6–54.5]
Verhoef et al.^63^	Cancer and ED	12	No	245	78.8	89.1 [83.9–92.8]	40.4 [28.2–53.9]
Edge et al.^64^ – 6 month timeframe	Cancer	6	Yes	1276	25.4	71.0 [65.8–75.7]	53.6 [50.4–56.7]
Edge et al.^64^ – 12 month timeframe	Cancer	12	Yes	655	42.4	68.7 [63.0–73.9]	63.7 [58.7–68.4]
Ernecoff et al.^65^	Kidney disease	12	Yes	95	9.5	66.7 [35.4–87.9]	75.6 [65.5–83.4]
Flierman et al.^66^	Hospital inpatients	12	No	252	30.6	81.8 [71.8–88.8]	48.6 [41.3–55.9]
Ikari et al.^67^	Cancer	0.1	No	1411	47.8	94.4 [92.4–95.9]	26.3 [23.3–29.6]
Estifan Kasabji et al.^68^	Kidney disease	12	Yes	178	15.7	71.4 [52.9–84.7]	64.7 [56.7–71.9]
Lai et al.^69^	Kidney disease	12	No	401	8.5	52.9 [36.7–68.5]	95.6 [93.0–97.3]
Maes et al.^70^ – subgroup 1	Hospital inpatients	12	No	185	24.9	67.4 [53.0–79.1]	78.4 [70.9–84.4]
Maes et al.^70^– subgroup 2	Cardiac disease	12	No	183	20.2	67.6 [51.5–80.4]	76.0 [68.5–82.2]
Rauh et al.^71^	Cancer	12	No	309	23.6	75.3 [64.4–83.8]	68.6 [62.5–74.2]
Tabernero Huguet et al.^72^	Pulmonary disease	12	Yes	361	23.5	65.9 [55.3–75.1]	86.6 [82.1–90.1]
Tak et al.^73^	Pulmonary disease	12	Yes	123	18.7	73.9 [53.5–87.5]	84.0 [75.6–89.9]
Valerio and Farinha^75^	Kidney disease	12	Yes	194	11.3	68.2 [47.3–83.6]	77.3 [70.5–82.9]
van Wijmen et al.^76^	Primary care	12	No	3640	1.0	50.0 [34.5–65.5]	98.6 [98.2–99.0]
Yen et al.^77^	Hospital inpatients	12	No	21,098	8.3	45.6 [43.2–47.9]	90.6 [90.2–91.0]
Ermers et al.^78^	Cancer	12	No	379	31.1	87.3 [80.1–92.1]	67.4 [61.5–72.8]
Tripp et al.^74^ – 1 month timeframe	Pulmonary disease	1	No	381	4.2	12.5 [3.5–36.0]	95.3 [92.7–97.1]
Tripp et al.^74^ – 12 month timeframe	Pulmonary disease	12	No	365	22.2	46.9 [36.4–57.7]	75.4 [70.0–80.0]

* Confidence intervals (CI) were calculated using Wilson’s method and can differ slightly from the CI’s presented by the original studies.

## References

[1] WHO. Palliative care, https://www.who.int/health-topics/palliative-care (accessed 18 May 2021).

[2] Lynn J Schall MW Milne C , et al. Quality improvements in end of life care: insights from two collaboratives. Jt Comm J Qual Improv 2000; 26: 254–267. 18350770 10.1016/s1070-3241(00)26020-3

[3] Downar J Goldman R Pinto R , et al. The ‘surprise question’ for predicting death in seriously ill patients: a systematic review and meta-analysis. CMAJ 2017; 189: E484–E493. 10.1503/cmaj.160775 PMC5378508 28385893

[4] White N Kupeli N Vickerstaff V , et al. How accurate is the ‘Surprise Question’ at identifying patients at the end of life? A systematic review and meta-analysis. BMC Med 2017; 15(1): 139. 28764757 10.1186/s12916-017-0907-4 PMC5540432

[5] Moher D Liberati A Tetzlaff J , et al. Preferred reporting items for systematic reviews and meta-analyses: the PRISMA statement. PLoS Med 2009; 6(7): e1000097. 10.1371/journal.pmed.1000097 PMC2707599 19621072

[6] Page MJ McKenzie JE Bossuyt PM , et al. The PRISMA 2020 statement: an updated guideline for reporting systematic reviews. Internet J Surg 2021; 88: 105906. 10.1016/j.ijsu.2021.105906 33789826

[7] Thomas K Noble B. Improving the delivery of palliative care in general practice: an evaluation of the first phase of the Gold Standards Framework. Palliat Med 2007; 21: 49–53. 17169960 10.1177/0269216306072501

[8] Gómez-Batiste X Martínez-Muñoz M Blay C , et al. Identifying patients with chronic conditions in need of palliative care in the general population: development of the NECPAL tool and preliminary prevalence rates in Catalonia. BMJ Support Palliat Care 2013; 3(3): 300–308. 10.1136/bmjspcare-2012-000211 24644748

[9] Ouzzani M Hammady H Fedorowicz Z , et al. Rayyan-a web and mobile app for systematic reviews. Syst Rev 2016; 5: 210. 27919275 10.1186/s13643-016-0384-4 PMC5139140

[10] Hayden JA Côté P Bombardier C. Evaluation of the quality of prognosis studies in systematic reviews. Ann Intern Med 2006; 144: 427–437. 16549855 10.7326/0003-4819-144-6-200603210-00010

[11] Wilson EB. Probable inference, the law of succession, and statistical inference. J Am Stat Assoc 1927; 22: 209–212.

[12] Reitsma JB Glas AS Rutjes AW , et al. Bivariate analysis of sensitivity and specificity produces informative summary measures in diagnostic reviews. J Clin Epidemiol 2005; 58: 982–990. 16168343 10.1016/j.jclinepi.2005.02.022

[13] Walter SD. Properties of the summary receiver operating characteristic (SROC) curve for diagnostic test data. Stat Med 2002; 21: 1237–1256. 12111876 10.1002/sim.1099

[14] Greene WH. Econometric analysis. 7th ed. Boston, MA: Prentice Hall, 2012.

[15] Higgins JPT Thompson SG . Quantifying heterogeneity in a meta-analysis. Stat Med 2002; 21: 1539–1558. 12111919 10.1002/sim.1186

[16] SAS ® Reference. The statistical analysis was performed with SAS statistical software version 9.4 (SAS Institute, North-Carolina, USA).

[17] Microsoft Corporation. Microsoft Excel [Internet], https://office.microsoft.com/excel (2018).

[18] Barnes S Gott M Payne S , et al. Predicting mortality among a general practice-based sample of older people with heart failure. Chronic Illn 2008; 4: 5–12. 18322025 10.1177/1742395307083783

[bibr19-02692163221099116] Faria de Sousa P Julião M Rodrigues A , et al. Accuracy of the Surprise Question on Patients with Advanced Chronic Disease in the Primary Care Setting 2019.

[20] Gibbins J Bloor S Reid C , et al. The use of a modified ‘surprise’ question to identify and recruit dying patients into a research project in an acute hospital setting. BMJ Support Palliat Care 2012; 2: A8.

[21] Moss AH Ganjoo J Sharma S , et al. Utility of the “Surprise” question to identify dialysis patients with high mortality. Clin J Am Soc Nephrol 2008; 3: 1379–1384. 18596118 10.2215/CJN.00940208 PMC2518805

[bibr22-02692163221099116] Cohen LM Ruthazer R Moss AH , et al. Predicting six-month mortality for patients who are on maintenance hemodialysis. Clin J Am Soc Nephrol 2010; 5: 72–79. 19965531 10.2215/CJN.03860609 PMC2801643

[bibr23-02692163221099116] Moss AH Lunney JR Culp S , et al. Prognostic significance of the “Surprise” question in cancer patients. J Palliat Med 2010; 13: 837–840. 20636154 10.1089/jpm.2010.0018

[bibr24-02692163221099116] South G Reddington O Hatfield L , et al. End of life in COPD: there may be no surprises! Eur Respir J 2011; 38: 1241.22045799

[bibr25-02692163221099116] Fenning S Woolcock R Haga K , et al. Identifying acute coronary syndrome patients approaching end-of-life. PLoS One 2012; 7: e35536. 10.1371/journal.pone.0035536 PMC3329478 22530044

[bibr26-02692163221099116] Da Silva Gane M Braun A Stott D , et al. How robust is the ‘surprise question’ in predicting short-term mortality risk in haemodialysis patients. Nephron Clin Pract 2013; 123: 185–193. 23921223 10.1159/000353735

[bibr27-02692163221099116] Pang W-F Kwan BC Chow K-M , et al. Predicting 12-month mortality for peritoneal dialysis patients using the ‘surprise’ question. Perit Dial Int 2013; 33: 60–66. 22855890 10.3747/pdi.2011.00204 PMC3598271

[bibr28-02692163221099116] Reilly L Reilly K McCloskey M , et al. Prognostic significance of the ‘surprise question’ in an respiratory inpatient population in a DGH. Ir J Med Sci 2013; 182: S484.

[bibr29-02692163221099116] Moroni M Zocchi D Bolognesi D , et al. The ‘surprise’ question in advanced cancer patients: a prospective study among general practitioners. Palliat Med 2014; 28: 959–964. 24662237 10.1177/0269216314526273

[bibr30-02692163221099116] Feyi K Klinger S Pharro G , et al. Predicting palliative care needs and mortality in end stage renal disease: use of an at-risk register. BMJ Support Palliat Care 2015; 5: 19–25. 10.1136/bmjspcare-2011-000165 24644161

[bibr31-02692163221099116] Hamano J Morita T Inoue S , et al. Surprise questions for survival prediction in patients with advanced cancer: a multicenter prospective cohort study. Oncologist 2015; 20: 839–844. 26054631 10.1634/theoncologist.2015-0015 PMC4492240

[bibr32-02692163221099116] Amro OW Ramasamy M Strom JA , et al. Nephrologist-facilitated advance care planning for hemodialysis patients: a quality improvement project. Am J Kidney Dis 2016; 68: 103–109. 26806003 10.1053/j.ajkd.2015.11.024 PMC4921274

[bibr33-02692163221099116] Maria Carmen J Santiago P Elena D , et al. Frailty, surprise question and mortality in a hemodilaysis cohort question and mortality in a hemodialysis cohort. Nephrol Dial Transplant 2016; 31: i553.

[bibr34-02692163221099116] Gerlach C Halbe L Goebel S , et al. The role of the “Surprise”-Question in hematooncology: “Would I be surprised if this patient died in the next 12 months?” Quantitative and qualitative analyses of a pilot project in the care of outpatients of an academic hospital in Germany. Palliat Med 2016; 30: S12.

[bibr35-02692163221099116] Lakin JR Robinson MG Bernacki RE , et al. Estimating 1-year mortality for high-risk primary care patients using the ‘Surprise’ question. JAMA Intern Med 2016; 176: 1863–1865. 10.1001/jamainternmed.2016.5928 PMC5755589 27695853

[bibr36-02692163221099116] Strout TD Haydar SA Eager E , et al. Identifying unmet palliative care needs in the ED: use of the ‘surprise question’ in patients with sepsis. Acad Emerg Med 2016; 23: S196.

[bibr37-02692163221099116] Gómez-Batiste X Martínez-Muñoz M Blay C , et al. Utility of the NECPAL CCOMS-ICO© tool and the surprise question as screening tools for early palliative care and to predict mortality in patients with advanced chronic conditions: a cohort study. Palliat Med 2017; 31: 754–763. 27815556 10.1177/0269216316676647

[bibr38-02692163221099116] Hadique S Culp S Sangani RG , et al. Derivation and validation of a prognostic model to predict 6-month mortality in an intensive care unit population. Ann Am Thorac Soc 2017; 14: 1556–1561. 28598196 10.1513/AnnalsATS.201702-159OC

[bibr39-02692163221099116] Lilley EJ Gemunden SA Kristo G , et al. Utility of the ‘Surprise’ question in predicting survival among older patients with acute surgical conditions. J Palliat Med 2017; 20: 420–423. 27802091 10.1089/jpm.2016.0313 PMC5385444

[bibr40-02692163221099116] Moretti C Iqbal J Murray S , et al. Prospective assessment of a palliative care tool to predict one-year mortality in patients with acute coronary syndrome. Eur Heart J Acute Cardiovasc Care 2017; 6: 272–279. 26880851 10.1177/2048872616633841

[bibr41-02692163221099116] Salat H Javier A Siew ED , et al. Nephrology provider prognostic perceptions and care delivered to older adults with advanced kidney disease. Clin J Am Soc Nephrol 2017; 12: 1762–1770. 28923833 10.2215/CJN.03830417 PMC5672972

[bibr42-02692163221099116] Santos Lascasas J Fonseca I Malheiro J , et al. Predicting six month mortality in elderly dialysis patients: a simple prognostic score. Nephrol Dial Transplant 2017; 32: iii346.

[bibr43-02692163221099116] Strout TD Haydar SA Vogt A. Identifying unmet palliative care needs: use and utility of the “surprise question” in emergency and inpatient settings. Acad Emerg Med 2017; 24: S149. 10.1089/jpm.2016.0403 28437203

[bibr44-02692163221099116] Burke K Coombes LH Menezes A , et al. The ‘surprise’ question in paediatric palliative care: a prospective cohort study. Palliat Med 2018; 32: 535–542. 28627303 10.1177/0269216317716061

[bibr45-02692163221099116] Ebke M Koch A Dillen K , et al. The ‘Surprise Question’ in neurorehabilitation—prognosis estimation by neurologist and palliative care physician; a longitudinal, prospective, observational study. Front Neurol 2018; 9: 792. 30319526 10.3389/fneur.2018.00792 PMC6165871

[bibr46-02692163221099116] Faria de Sousa P Julião M Rodrigues AP , et al. Accuracy of the Surprise Question on Patients with Advanced Chronic Disease in the Primary Care Setting: Preliminary Results. J Palliat Med 2018; 21: 410–411.

[bibr47-02692163221099116] Forzley B Er L Chiu HHL , et al. External validation and clinical utility of a prediction model for 6-month mortality in patients undergoing hemodialysis for end-stage kidney disease. Palliat Med 2018; 32: 395–403. 28731382 10.1177/0269216317720832 PMC5788083

[bibr48-02692163221099116] Liyanage T Mitchell G Senior H. Identifying palliative care needs in residential care. Aust J Prim Health 2018; 24: 524–529. 30423282 10.1071/PY17168

[bibr49-02692163221099116] Mitchell GK Senior HE Rhee JJ , et al. Using intuition or a formal palliative care needs assessment screening process in general practice to predict death within 12 months: a randomised controlled trial. Palliat Med 2018; 32: 384–394. 28452570 10.1177/0269216317698621

[bibr50-02692163221099116] Mudge AM Douglas C Sansome X , et al. Risk of 12-month mortality among hospital inpatients using the surprise question and SPICT criteria: a prospective study. BMJ Support Palliat Care 2018; 8: 213–220. 10.1136/bmjspcare-2017-001441 29500239

[bibr51-02692163221099116] Ouchi K Jambaulikar G George NR , et al. The ‘Surprise Question’ asked of emergency physicians may predict 12-month mortality among older emergency department patients. J Palliat Med 2018; 21: 236–240. 28846475 10.1089/jpm.2017.0192 PMC6909689

[bibr52-02692163221099116] Schmidt RJ Landry DL Cohen L , et al. Derivation and validation of a prognostic model to predict mortality in patients with advanced chronic kidney disease. Nephrol Dial Transplant 2019; 34: 1517–1525. 30395311 10.1093/ndt/gfy305

[bibr53-02692163221099116] Tanasiychuk T Antebi A Kushnir D , et al. Prognostic tool in dialysis treated AKI. J Am Soc Nephrol 2018; 29: 876–877.

[bibr54-02692163221099116] Aaronson EL George N Ouchi K , et al. The surprise question can be used to identify heart failure patients in the emergency department who would benefit from palliative care. J Pain Symptom Manag 2019; 57: 944–951. 10.1016/j.jpainsymman.2019.02.007 PMC6713219 30776539

[bibr55-02692163221099116] Gastelurrutia P Zamora E Domingo M , et al. Palliative care needs in heart failure. A multicenter study using the NECPAL Questionnaire. Rev Esp Cardiol 2019; 72: 870–872. 30850349 10.1016/j.rec.2019.01.009

[bibr56-02692163221099116] Haydar SA Strout TD Bond AG , et al. Prognostic value of a modified surprise question designed for use in the emergency department setting. Clin Exp Emerg Med 2019; 6: 70–76. 30944292 10.15441/ceem.17.293 PMC6453688

[bibr57-02692163221099116] Lakin JR Robinson MG Obermeyer Z , et al. Prioritizing primary care patients for a communication intervention using the ‘Surprise Question’: a prospective cohort study. J Gen Intern Med 2019; 34: 1467–1474. 31190257 10.1007/s11606-019-05094-4 PMC6667512

[bibr58-02692163221099116] Ouchi K Strout T Haydar S , et al. Association of emergency clinicians’ assessment of mortality risk with actual 1-month mortality among older adults admitted to the hospital. JAMA Netw Open 2019; 2: e1911139. 10.1001/jamanetworkopen.2019.11139 PMC6745053 31517962

[bibr59-02692163221099116] Raubenheimer PJ Day C Abdullah F , et al. The utility of a shortened palliative care screening tool to predict death within 12 months - a prospective observational study in two South African hospitals with a high HIV burden. BMC Palliat Care 2019; 18: 101. 31722691 10.1186/s12904-019-0487-5 PMC6854790

[bibr60-02692163221099116] Straw S Byrom R Gierula J , et al. Predicting one-year mortality in heart failure using the ‘Surprise Question’: a prospective pilot study. Eur J Heart Fail 2019; 21: 227–234. 30548129 10.1002/ejhf.1353

[bibr61-02692163221099116] Tripodoro VA Llanos V De Lellis S , et al. Prognostic factors in cancer patients with palliative needs identified by the NECPAL CCOMS-ICO© tool. Medicina 2019; 79: 95–103. 31048274

[bibr62-02692163221099116] Veldhoven CMM Nutma N De Graaf W , et al. Screening with the double surprise question to predict deterioration and death: an explorative study. BMC Palliat Care 2019; 18(1): 118. 31881958 10.1186/s12904-019-0503-9 PMC6935168

[bibr63-02692163221099116] Verhoef M-J de Nijs EJM Fiocco M , et al. Surprise question and performance status indicate urgency of palliative care needs in patients with advanced cancer at the emergency department: an observational cohort study. J Palliat Med 2020; 23: 801–808. 31880489 10.1089/jpm.2019.0413

[bibr64-02692163221099116] Edge SB Liu L Case AA , et al. Value of oncologist generated “surprise question” in predicting survival in metastatic cancer. J Clin Oncol 2020; 38: 12082.

[bibr65-02692163221099116] Ernecoff NC Abdel-Kader K Cai M , et al. Implementation of surprise question assessments using the electronic health record in older adults with advanced CKD. J Am Soc Nephrol 2020; 31: 224. 10.34067/KID.0007062020 PMC8791363 35373084

[bibr66-02692163221099116] Flierman I van Rijn M Willems DL , et al. Usability of the surprise question by nurses to identify 12-month mortality in hospitalized older patients: a prospective cohort study. Int J Nurs Stud 2020; 109: 103609. 32603926 10.1016/j.ijnurstu.2020.103609

[bibr67-02692163221099116] Ikari T Hiratsuka Y Yamaguchi T , et al. ‘3-Day Surprise Question’ to predict prognosis of advanced cancer patients with impending death: multicenter prospective observational study. Cancer Med 2021; 10: 1018–1026. 33347734 10.1002/cam4.3689 PMC7897938

[bibr68-02692163221099116] Estifan Kasabji J Lucas C Sastre A , et al. Is the surprise question useful as a predictor of mortality in hemodialysis patients? Nephrol Dial Transplant 2020; 35: iii1452.

[bibr69-02692163221099116] Lai C-F Cheng C-I Chang C-H , et al. Integrating the surprise question, palliative care screening tool, and clinical risk models to identify peritoneal dialysis patients with high one-year mortality. J Pain Symptom Manag 2020; 60(3): 613–621.e6. 10.1016/j.jpainsymman.2020.03.035 32278098

[bibr70-02692163221099116] Maes H Van Den Noortgate N De Brauwer I , et al. Prognostic value of the surprise question for one-year mortality in older patients: a prospective multicenter study in acute geriatric and cardiology units. Acta Clin Belg 2022; 77: 286–294. 33044915 10.1080/17843286.2020.1829869

[bibr71-02692163221099116] Rauh LA Sullivan MW Camacho F , et al. Validation of the surprise question in gynecologic oncology: a one-question screen to promote palliative care integration and advance care planning. Gynecol Oncol 2020; 157: 754–758. 32171568 10.1016/j.ygyno.2020.03.007

[bibr72-02692163221099116] Tabernero Huguet E Ortiz de Urbina Antia B González Quero B , et al. Prevalence and mortality of patients with palliative needs in an acute respiratory setting. Arch Bronconeumol 2021; 57: 729. 35699026 10.1016/j.arbr.2021.09.015

[bibr73-02692163221099116] Tak N Moor C Owusuaa C , et al. The value of the surprise question to predict mortality in idiopathic pulmonary fibrosis. Eur Respir J 2020; 56: 1800. 10.1159/000516291 PMC8491469 34044401

[bibr74-02692163221099116] Tripp D Janis J Jarrett B , et al. How well does the surprise question predict 1-year mortality for patients admitted with COPD? J Gen Intern Med 2021; 36: 2656–2662. 33409886 10.1007/s11606-020-06512-8 PMC8390592

[bibr75-02692163221099116] Valerio P Farinha A. Surprise question: a mortality predictor in hemodialysis patients? J Am Soc Nephrol 2020; 31: 407.

[bibr76-02692163221099116] van Wijmen MPS Schweitzer BPM Pasman HR , et al. Identifying patients who could benefit from palliative care by making use of the general practice information system: the surprise question versus the SPICT. Fam Pract 2020; 37: 641–647. 32424418 10.1093/fampra/cmaa049 PMC7571774

[bibr77-02692163221099116] Yen Y-F Lee Y-L Hu HY , et al. Early palliative care: the surprise question and the palliative care screening tool—better together. BMJ Support Palliat Care. Epub ahead of print 25 May 2020. DOI: 10.1136/bmjspcare-2019-002116. 32451326

[78] Ermers DJ Kuip EJ Veldhoven C , et al. Timely identification of patients in need of palliative care using the double surprise question: a prospective study on outpatients with cancer. Palliat Med 2021; 35: 592–602. 33423610 10.1177/0269216320986720 PMC7975860

[79] Milnes S Orford NR Berkeley L , et al. A prospective observational study of prevalence and outcomes of patients with gold standard framework criteria in a tertiary regional Australian Hospital. BMJ Support Palliat Care 2019; 9: 92–99. 10.1136/bmjspcare-2015-000864 26391750

[80] Ramsenthaler C Haberland B Schneider S , et al. Identifying patients with cancer appropriate for early referral to palliative care using the integrated palliative care outcome scale (IPOS)-a cross-sectional study of acceptability and deriving valid cut-points for screening. Palliat Med 2018; 32: 98.

[81] Haga K Murray S Reid J , et al. Identifying community based chronic heart failure patients in the last year of life: a comparison of the Gold Standards Framework Prognostic Indicator Guide and the Seattle Heart Failure Model. Heart 2012; 98: 579–583. 22422744 10.1136/heartjnl-2011-301021

[82] O’Callaghan A Laking G Frey R , et al. Can we predict which hospitalised patients are in their last year of life? A prospective cross-sectional study of the Gold Standards Framework Prognostic Indicator Guidance as a screening tool in the acute hospital setting. Palliat Med 2014; 28: 1046–1052. 24854032 10.1177/0269216314536089

[83] Vick JB Pertsch N Hutchings M , et al. The utility of the surprise question in identifying patients most at risk of death. J Clin Oncol 2015; 33: 8.

[84] Zertuche-Maldonado T Tellez-Villarreal R Pascual A , et al. Palliative care needs in an acute internal medicine ward in Mexico. J Palliat Med 2018; 21: 163–168. 28846483 10.1089/jpm.2017.0043

[85] Kittanamongkolchai W Suarez MLG Gregoire JR. Dialysis: palliative and end-of-life care external validation of a short-term prognostic model for patients who are on maintenance hemodialysis. J Am Soc Nephrol 2017; 28: 638.

[86] Gillespie S Lane ND Echevarria C , et al. The ‘surprise question’ in patients surviving severe COPD exacerbation. Eur Respir J 2020; 56: 109.

[87] Sangani R Mokaya E Mujahid H , et al. Early high-risk patient identification and palliative care intervention does not lead to self-fulfilling prophecy in the ICU. Chest 2020; 158: A1849.

[88] Sangani R Mokaya E Mujahid H , et al. Early palliative care intervention reduces ICU readmissions in high-risk patients. Chest 2020; 158: A1841.

[89] Duenk RG Verhagen C Bronkhorst EM , et al. Development of the ProPal-COPD tool to identify patients with COPD for proactive palliative care. Int J Chron Obstruct Pulmon Dis 2017; 12: 2121–2128. 28790815 10.2147/COPD.S140037 PMC5530053

[90] Rice J Hunter L Hsu AT , et al. Using the ‘Surprise Question’ in nursing homes: a prospective mixed-methods study. J Palliat Care 2018; 33: 9–18. 29260612 10.1177/0825859717745728

[91] Carvalho JR Vasconcelos M Marques da Costa P , et al. Identifying palliative care needs in a Portuguese liver unit. Liver Int 2018; 38: 1982–1987. 29682885 10.1111/liv.13865

[92] Gaffney L Judge C Morrison L , et al. Use of the ‘Surprise Question’ in predicting adverse outcomes among frail older patients after hospital admission. Age Ageing 2018; 47: v1–v12.

[93] Glick J Marin BG Chelluri J , et al. Utility of the” surprise question” in critically ill emergency department patients. Ann Emerg Med 2018; 72: S68.

[94] Singh S Graham Z Rodriguez A , et al. Accuracy of the surprise question on an inpatient oncology service: a multidisciplinary perspective. J Hosp Palliat Nurs 2019; 21: 300–304. 30933015 10.1097/NJH.0000000000000558

[95] Li DY Prigmore HL Stewart TG , et al. Development and internal validation of a mortality risk prediction model in older adults with advanced non-dialysis-dependent (NDD) CKD. J Am Soc Nephrol 2020; 31: 213.

[96] Thiagarajan R Morris J Harkins KJ. Can simple intuitive questions identify patients in the last year of their life?-a pragmatic study comparing the “paired surprise questions” with the “single surprise question”. Age Ageing 2012; 41: i61.

[97] Lledo MD Ahumada M Puche AM , et al. Palliative care in cardiological patients, a forgotten problem. Eur Heart J 2014; 35: 835–836.

[98] Lefkowits C Chandler C Sukumvanich P , et al. Validation of the “surprise question” in gynecologic oncology: comparing physicians, advanced practice providers and nurses. Gynecol Oncol 2016; 141: 128.26867989

[99] Gopinathan J Aboobacker I Hafeeq B , et al. Predicting death on maintenance hemodialysis-a complex task in prevalent elders. Nephrol Dial Transplant 2016; 31: i550.

[100] Wong J Gott M Frey R , et al. Palliative care presentations to emergency departments in a secondary and a sub-acute hospital: a one year incidence study. Prog Palliat Care 2017; 25: 235–241.

[101] Mastandrea M Rojas L Costa D , et al. Frailty and functional status in elderly patients with acute coronary syndrome: prospective cohort study to assess mortality risk. Eur Heart J 2018; 39: 704.

[102] Rubinfeld G Boodram P Ho Cho M , et al. The prognostic accuracy of the ‘surprise question’ in geriatric patients at a large New York City hospital. J Am Geriatr Soc 2019; 67: S290.

[103] De La Puente MÍNM Del Rosario Evangelista Cabrera L Mendoza SD , et al. Advanced chronic disease and palliative needs in an acute geriatric unit. Eur Geriatr Med 2019; 10: S49.

[104] Ahmed I Sutton AJ Riley RD. Assessment of publication bias, selection bias, and unavailable data in meta-analyses using individual participant data: a database survey. BMJ 2012; 344: d7762. 10.1136/bmj.d7762 22214758

[105] Elliott M Nicholson C. A qualitative study exploring use of the surprise question in the care of older people: perceptions of general practitioners and challenges for practice. BMJ Support Palliat Care 2017; 7: 32–38. 10.1136/bmjspcare-2014-000679 25168076

[106] Haydar SA Almeder L Michalakes L , et al. Using the surprise question to identify those with unmet palliative care needs in emergency and inpatient settings: what do clinicians think? J Palliat Med 2017; 20: 729–735. 28437203 10.1089/jpm.2016.0403

[107] De Bock R Van Den Noortgate N Piers R . Validation of the supportive and palliative care indicators tool in a geriatric population. J Palliat Med 2018; 21(2): 220–224. 28792787 10.1089/jpm.2017.0205

[108] Cardona-Morrell M Kim J Turner RM , et al. Non-beneficial treatments in hospital at the end of life: a systematic review on extent of the problem. Int J Qual Health Care 2016; 28: 456–469. 27353273 10.1093/intqhc/mzw060

[109] Brinkman-Stoppelenburg A Rietjens JA van der Heide A. The effects of advance care planning on end-of-life care: a systematic review. Palliat Med 2014; 28: 1000–1025. 24651708 10.1177/0269216314526272

[110] Billings JA Bernacki R. Strategic targeting of advance care planning interventions: the Goldilocks phenomenon. JAMA Intern Med 2014; 174: 620–624. 24493203 10.1001/jamainternmed.2013.14384

[111] Walsh RI Mitchell G Francis L , et al. What diagnostic tools exist for the early identification of palliative care patients in general practice? A systematic review. J Palliat Care 2015; 31: 118–123. 26201214 10.1177/082585971503100208

